# Mechanism of Babao Dan on fluorouracil-induced bone marrow suppression and T cell immune disorder through MAPK pathway

**DOI:** 10.1038/s41598-026-35751-8

**Published:** 2026-01-12

**Authors:** Shuo Yan, Ruihan Sun, Jie Yuan, Peizhi Jia, Ruiming Yang, Jiajun He, Junying Guo, Yun Liu, Jinhong Liu, Xuzheng Chen, Minghe Lin, Jiumao Lin

**Affiliations:** 1 People’s Hospital Affiliated to Fujian University of Traditional Chinese Medicine, Fuzhou, 350004 Fujian Province China; 2 Academy of Integrative Medicine, Fujian University of Traditional Chinese Medicine, Fuzhou, 350122 Fujian Province China; 3 Fujian Key Laboratory of Integrative Medicine on Geriatrics, Fuzhou, 350122 Fujian Province China; 4 College of Integrative Medicine, Fujian University of Traditional Chinese Medicine, Fuzhou, 350122 Fujian Province China; 5 Academy of Pharmacy, Fujian University of Traditional Chinese Medicine, Fuzhou, 350122 Fujian China; 6 Fujian Medical University Cancer Hospital, Fuzhou, 350014 Fujian China; 7 Fujian Cancer Hospital, Fuzhou, 350014 Fujian China

**Keywords:** BBD, 5-FU, Myelosuppression, T lymphocyte subsets, MAPK signaling pathway, Cancer, Cell biology, Immunology

## Abstract

Babao Dan (BBD), a traditional Chinese herbal compound, has demonstrated significant antitumor effects and is clinically used as an adjunctive therapy for various gastrointestinal malignancies. However, its underlying mechanisms of action remain poorly understood. This study aimed to investigate the effects of BBD on 5-fluorouracil (5-FU)-induced myelosuppression and T-lymphocyte subpopulation alterations, and to explore its mechanism in ameliorating cellular immune dysfunction caused by myelosuppression via the MAPK signaling pathway. The major constituents of BBD are fatty acids, triterpenoids, flavonoids, and alkaloids, along with other compounds. BBD significantly improved survival, reduced body weight loss, and mitigated the decline in splenic index in mice following 5-FU-induced chemotherapy. Additionally, it alleviated the reduction of peripheral blood cells, including leukocytes, neutrophils, and reticulocytes, and attenuated 5-FU-induced bone marrow hematopoiesis suppression. Furthermore, BBD restored the imbalance in the proportions of Th1 and Th2 cells within the spleens of 5-FU-induced mice. It also reversed the inhibition of CD25^+^ and CD69^+^ expression, the reduction in lymphocyte division generation, and the decreased expression of Ki67 and PCNA, thereby enhancing T-cell activation and proliferation in splenic cells. Moreover, BBD alleviated the G1 phase blockage in the bone marrow cell cycle and upregulated the expression of key proteins, including CDK2, CDK4, CDK6, Cyclin D1, Cyclin E1, p-Rb, p-c-Fos, p-c-Jun, p-ERK, p-NF-κB p65, and p-p38, in bone marrow cells. BBD may promote the expression of G1/S checkpoint-related proteins in myeloid cells by regulating the MAPK signaling pathway, thereby upregulating the phosphorylation level of Rb and facilitating the transition from the G1 phase to the S phase. This orderly cell cycle progression enhances cell proliferation and improves the hematopoietic function of myeloid cells after 5-FU chemotherapy. Consequently, BBD restores the ratio of peripheral T-cell subsets, corrects the imbalance of Th-cell subsets, and enhances the activation and proliferation functions of CD4^+^T cells and CD8^+^T cells, ultimately rectifying the dysregulated immune homeostasis of the organism.

## Introduction

According to recent statistics, China projected approximately 4,824,700 new cancer cases and 2,574,200 cancer-related deaths in 2022, posing a significant public health burden^1^. Chemotherapy remains a primary treatment for malignant tumors, effectively inhibiting tumor cell proliferation and metastasis. However, its non-selective cytotoxicity also damages healthy cells, leading to adverse effects such as nausea, vomiting, leukopenia, and bone marrow suppression. Severe myelosuppression compromises hematopoietic and immune function, often forcing treatment discontinuation due to poor tolerance. This increases infection risks and may contribute to tumor recurrence or metastasis, ultimately worsening patient prognosis^2–4^.

Research indicates that chemotherapy can impact peripheral blood T lymphocyte subsets by compromising bone marrow hematopoietic cells and thymic microenvironment. This disruption leads to an imbalance or functional inactivation of peripheral blood T lymphocyte subsets within the organism. Alterations in the quantity and functionality of these lymphocyte subsets have been correlated with the overall survival (OS) of cancer patients, thereby directly influencing their prognosis^5–7^. Bone marrow is essential for the synthesis of various cytokines and growth factors, which are integral to the activation of T cells. Chemotherapy, for instance, can lead to a reduction in the production of cytokines such as IFN-γ, IL-7, IL-12, and IL-21 within the bone marrow hematopoietic microenvironment. This reduction impairs the capacity of hematopoietic cells to recruit T cells and adversely affects the proliferation and activation of T cells^8^. Chemotherapy exposes the bone marrow to a pathologically hypoxic microenvironment, which can induce bone marrow mesenchymal stem cells (MSCs) to inhibit the proliferation of activated CD4^+^T cells through a transforming growth factor-beta (TGF-β) and IL-10-dependent autophagic process. In addition, bone marrow progenitor cell-derived dendritic cells (DCs) are key antigen-presenting cells in the organism, and they have a direct impact on the immunoregulatory capacity of CD8^+^T cells. Chemotherapy affects the antigen-presenting function of DCs by destroying the function of bone marrow progenitor cells, which makes antigens ineffective in being recognised by CD8^+^T cells, and affects the killing function of CD8^+^T cells^9–11^.This shows that improving chemotherapy-induced myelosuppression and thus restoring bone marrow haematopoiesis is an important way to improve T-cell immunosuppression in tumour patients after chemotherapy.

Babao Dan (BBD), a nationally protected Traditional Chinese Medicine (TCM), has demonstrated multifaceted therapeutic effects in our previous studies. Clinically, BBD can be applied in combination therapy for the treatment of pancreatic cancer^12^. Initially, we demonstrated its ability to alleviate 5-fluorouracil (5-FU)-induced toxicity, reducing weight loss, diarrhea, leukopenia, thrombocytopenia, and colonic shortening while inhibiting jejunal apoptosis and promoting tissue proliferation—thereby preserving intestinal mucosal integrity^13,14^. Beyond chemotherapy protection, BBD also ameliorates cancer cachexia in mice^15^. Notably, its therapeutic potential extends to direct antitumor effects: BBD suppresses the proliferation of well-differentiated cancer cells and disrupts the stem-like properties of cancer stem cells (CSCs) by modulating EpCAM expression^16^. Furthermore, it potently inhibits the migration, invasion, and lymphangiogenesis of gastric cancer cells^17,18^, collectively highlighting its dual role as both a chemoprotective agent and an antitumor therapy.

The impact of BBD on myelosuppression and immune dysregulation after 5-FU chemotherapy remains poorly understood. This study investigates BBD’s effects on 5-FU-induced myelosuppression, T lymphocyte subset alterations, and functionality, focusing on the MAPK signaling pathway’s role in hematopoietic processes. We aim to elucidate how BBD ameliorates immune dysregulation caused by myelosuppression, providing experimental evidence for its use during tumor chemotherapy. Our findings suggest that BBD modulates the MAPK pathway, upregulating G1/S checkpoint proteins and Rb phosphorylation, facilitating G1-to-S phase transition, and promoting cell proliferation. This restores hematopoietic function, corrects peripheral T cell subset imbalances, and enhances CD4^+^T cells and CD8^+^T cells activation and proliferation, ultimately alleviating immune dysregulation.

## Materials and methods

### Preparation of BBD

BBD (Lot No. 190330-B 190730) was obtained and authenticated by Xiamen Traditional Chinese Medicine Co., Ltd. (Xiamen, China). Based on clinical usage, the adult dose of BBD is 1.8 g daily. In accordance with the established equivalent dose conversion principle from humans to mice, where the mouse dose is estimated to be 9-fold that of the adult human dose, the BBD administration dose for mice was determined to be 250 mg/kg. The BBD powder was dissolved in physiological saline to achieve a concentration of 31.25 mg/mL. Fresh stock solution was prepared every 3 days, stored at 4 °C, and used within the 3‑day period. Before each administration, the solution was vortexed to ensure homogeneity.

### BBD non-targeted metabolomics analysis

A 50 mg sample was weighed, ground, and mixed with 500 µL of extraction solution (methanol: acetonitrile: water = 2: 2: 1), which contained isotope-labeled internal standards. After thorough mixing, the mixture was placed in a -40 °C refrigerator for 30 min. Then, it was centrifuged at 4 °C and 12,000 rpm for 15 min. The supernatant was collected and filtered through a 0.22 μm microporous membrane into an injection vial. The components in the powder were analyzed by non-targeted metabolomics using a Vanquish ultra-high-performance liquid chromatograph (Thermo Fisher Scientific, USA) equipped with a Phenomenex Kinetex C18 column (2.1 mm × 100 mm, 2.6 μm). The raw data were converted to the mzXML format using ProteoWizard and processed with an in-house program. The R package and the BiotreeDB (V3.0) were applied in metabolite identification.

### Materials and reagents

5-Fluorouracil (5-FU, A4071), proliferating cell nuclear antigen (PCNA) (D3H8P) XP^®^ Rabbit mAb (13110T), Ki-67 (D3B5) Rabbit mAb (12202T), p38 MAPK (D13E1) XP^®^ Rabbit mAb (8690), β-Tubulin Antibody (2146), Rb (D20) Rabbit mAb (9313), Phospho-Rb (Ser807/811) (D20B12) XP^®^ Rabbit mAb (8516), c-Fos (9F6) Rabbit mAb (2250), Phospho-c-Fos (Ser32) (D82C12) XP^®^ Rabbit mAb (5348), c-Jun (60A8) Rabbit mAb (9165), Phospho-c-Jun (Ser73) (D47G9) XP^®^ Rabbit mAb (3270), p44/42 MAPK (Erk1/2) (137F5) Rabbit mAb (4695), Phospho-p44/42 MAPK (Erk1/2) (Thr202/Tyr204) XP^®^ Rabbit mAb (4370), NF-κB p65 (D14E12) XP^®^ Rabbit mAb (8242), Phospho-NF-κB p65 (Ser536) (93H1) Rabbit mAb (3033), and Phospho-p38 MAPK (Thr180/Tyr182) (D3F9) XP^®^ Rabbit mAb (4511) were purchased from Cell Signaling Technology (Beverly, MA, USA). APC-Cy7 Rat Anti-Mouse CD45 (557659), FITC Hamster Anti-Mouse CD3e (553061), APC Hamster Anti-Mouse CD3e (553066), PE-CF594 Rat Anti-Mouse CD49b (562453), Alexa Fluor700 Rat Anti-Mouse CD8a (557959), BV786 Rat Anti-Mouse CD45R/B220 (563894), PE Rat Anti-Mouse F4/80 (565410), BV650 Rat Anti-Mouse Ly-6G (740554), BV421 Rat Anti-Mouse Ly-6 C (562727), PE Rat Anti-Mouse IL-17 A (559502), Leukocyte Activation Cocktail (550583), Fixable Viability Stain 510 (564406), PerCP-Cy™5.5 Rat Anti-Mouse CD8a (551162), BD Cytofix/Cytoperm™ Fixation/Permeabilization Kit (554714), PE Rat Anti-Mouse IFN-γ (554412), PE Rat Anti-Mouse IL-4 (554435), APC Rat Anti-Mouse CD25 (557192), FITC Rat Anti-Mouse CD4 (553650), Transcription Factor Buffer Set (562574), PE Rat anti-Mouse Foxp3 (563101), BV605 Rat Anti-Mouse CD4 (563151), BV421 Hamster Anti-Mouse γδ T-Cell Receptor (562892), PE Rat Anti-Mouse CD62L (553151), BV786 Rat Anti-Mouse CD44 (563736), BV605 Rat Anti-Mouse CD4 (563151), and PerCP-Cy™5.5 Rat Anti-Mouse CD69 (551113) were purchased from BD (New Jersey, USA). Dynabeads Mouse T-Activator CD3/CD28 (11452) was purchased from Gibco (Grand Island, USA). CD4^+^T Cell Isolation Kit (130-106-643), CD8^+^T Cell Isolation Kit (130-104-075) were purchased from Miltenyi Biotec (Bergisch Gladbach, Germany). GAPDH Monoclonal Antibody (60004-1-Ig), HRP-Goat Anti-Mouse IgG (H + L) (SA00001-1), HRP-Goat Anti-Rabbit IgG (H + L) (SA00001-2), Cyclin D1 Monoclonal antibody (60186-1-Ig) were purchased from Proteintech Group, Inc (Wuhan, Hubei, China). Rabbit Anti-CDK2 antibody (bs-10726R), Rabbit Anti-Cyclin E1 antibody (bs-0573R) were purchased from Bioss (Beijing, China). CDK4 Polyclonal Antibody (YT5198), CDK6 Polyclonal Antibody (YT5884) were purchased from ImmunoWay Biotechnology Company (Texas, USA). Methanol (67-56-1), Isopropanol (67-63-0) and acetonitrile (75-05-8) were purchased from CNW Technologies (Shanghai, China). Hydrochloric acid (7647-01-0) was purchased from Titan (Shanghai, China). Sodium chloride (7647-14-5) was purchased from Sangon Biotech (Shanghai, China). Acetic acid (64-19-7) was purchased from SIGMA-ALDRICH (Missouri, USA).

### Animals

The 5-FU dosing regimen was designed to achieve distinct experimental objectives: a higher dose (50 mg/kg) for survival analysis, and a lower dose (30 mg/kg) for detailed mechanistic and phenotypic investigations. In a mouse mortality observation experiment, 48 SPF-grade 8-week-old male BALB/c mice (weighing approximately 24–26 g; purchased from Shanghai SLAC Laboratory Animal Co., Ltd.) were randomly assigned into four groups (*n* = 12 per group): Control (Cont, saline via oral gavage), BBD (250 mg/kg via oral gavage, once daily for 15 days), 5-FU (50 mg/kg i.p., once daily for 5 days), and 5-FU + BBD (concurrent 5-FU i.p. and oral BBD at the aforementioned doses and schedules). Randomization was performed using the standard RAND() function in Microsoft Excel. Mortality was recorded throughout the experimental period. In a separate experiment where the dose of 5-FU was reduced to 30 mg/kg (with the same administration schedule), 28 BALB/c mice were randomly divided into four groups (*n* = 7 per group). Biological samples were collected at the end of the 15-day study for subsequent analysis.

For each animal, responsibilities were allocated as follows: a single investigator, who was solely aware of the group allocations, administered treatments according to the randomization list. All other investigators, who remained blinded to the treatment conditions, performed the assessments and measurements of all outcome indicators.

All animal experiments were conducted in accordance with the national guidelines for the humane treatment of animals and were approved by the Ethics Committee of Fujian University of Traditional Chinese Medicine (FJTCMIACUC 2023117). To ensure animal welfare: invasive procedures were performed under isoflurane anesthesia; and mice were euthanized under deep isoflurane anesthesia. In addition, all the study was carried out in compliance with the ARRIVE guidelines.

### Physiological status monitoring and spleen index analysis

Daily physiological monitoring was conducted on the general condition of the mice, encompassing survival rates and body weight changes. All mice underwent aseptic splenectomy, followed by photography and weight measurement. The spleen index (%) for each mouse was calculated using the formula: spleen weight (g) / mouse body weight (g) × 100. The spleen tissues were then preserved in RPMI-1640 medium for further analysis.

### Hematological analysis

Peripheral blood samples were collected from the mice via orbital venous sinus puncture using EDTA-anticoagulant tubes. Complete blood count analysis, including reticulocyte percentage (RET%) and reticulocyte maturity index (RMI), was performed using a standardized hematological analyzer (XN-A1, Sysmex, Kobe, Japan) at the Clinical Laboratory Center of Fujian Cancer Hospital.

### Reticulocyte staining and morphological observation

Reticulocyte morphological observation was performed using Brilliant Cresyl Blue (BCB) vital staining. Briefly, 10 µL of EDTA-anticoagulated whole blood was thoroughly mixed with an equal volume of Janus Green B staining solution (1:1 v/v). The mixture was incubated at room temperature for 10–15 min before preparing a blood smear. After staining, the smears were observed under a light microscope.

### Bone marrow cytology smears

The left femur of the mouse was dissected, and the bone marrow tissue was quickly flushed out onto an adhesive glass slide to create a bone marrow smear. The smear was then stained using the Wright-Giemsa method, and the cell morphology of bone marrow was observed under an oil immersion lens.

### Analysis of the proportions of Splenic leukocyte subsets

Splenocytes were mechanically dissociated by passing through a 70-µm cell strainer and washed with ice-cold PBS. The cell density was adjusted to 2 × 10^6^ cells/mL. Cells were resuspended in 500 µL PBS, stained with 0.5 µL Fixable Viability Stain 510, and incubated in the dark at room temperature for 10 min. After washing with Stain Buffer (FBS), the cells were resuspended in 100 µL of FBS and stained with 1 µg each of APC-Cy7-CD45, APC-CD3, PE-CF594-CD49b, BV786-CD45R/B220, Alexa Fluor700-CD8, PE-F4/80, BV421-Ly-6 C, and BV650-Ly-6G antibodies for 20 min in the dark. Flow cytometry analysis was performed following the staining procedure.

### Analysis of the proportions of T-cell subsets in peripheral blood

100 µL anticoagulated blood was stained with 0.1 µL of Fixable Viability Stain 510 and incubated in the dark at room temperature for 10 min. Following incubation, cells were washed with FBS and pelleted by centrifugation. The pellet was resuspended in 100 µL FBS and stained with a cocktail of 1 µg each of FITC-CD3, BV605-CD4, Alexa Fluor700-CD8, BV421-γδ TCR, BV786-CD44, and PE-CD62L antibodies for 20 min in the dark. Red blood cells were lysed, and the remaining cells washed twice with PBS. Finally, the proportions T-cell subsets were analyzed by flow cytometry.

### Analysis of the proportions of Th1, Th2, Th17 within the spleen

Splenocytes were treated with 2 µL Leukocyte Activation Cocktail (containing PMA, ionomycin, and brefeldin A) and incubated at 37 °C for 5 h. After PBS washing, cells were stained with Fixable Viability Stain 510 and incubated in the dark at room temperature for 10 min, then washed with FBS, stained with 1 µg each of APC-CD3 and PerCP-Cy5.5-CD8 antibodies, and incubated in the dark at room temperature for another 20 min. After washing with FBS, the cells were resuspended in 250 µL fixation/permeabilization solution, vortexed, and incubated in the dark at 4 °C for 20 min. Post-fixation, the cells were washed with Perm/Wash Buffer and stained with 1 µg each of PE-IFN-γ, PE-IL-4, and PE-IL-17 antibodies in separate tubes at 4 °C in the dark for 1.5 h. Finally, the cells were washed and prepared for flow cytometry analysis.

### Analysis of the proportions of Treg within the spleen

Cells were obtained from a spleen cell suspension in a quantity of 2 × 10^6^. They were washed and stained with 0.5 µL of Fixable Viability Stain 510 for 10 min in the dark at room temperature. After washing, cells were incubated with 1 µg each of FITC-CD4 and APC-CD25 antibodies for 20 min at room temperature in the dark. Following another wash, cells were fixed with 2 mL Fix/Perm Buffer for 50 min at 4 °C in the dark. After fixation, cells were stained with 1 µg PE-Foxp3 antibody for 2 h at 4 °C in the dark. Finally, cells were washed and analyzed by flow cytometry.

### Analysis of CD25 and CD69 expression in T cells

Splenocytes were plated at 2 × 10^6^ cells/mL in a 24-well plate with 25 µL Dynabeads Mouse T-Activator CD3/CD28 and 10 ng/mL IL-2 for 6 hours. After incubation, cellular activation was assessed, and dynabeads were removed using a magnetic rack. The cells were washed, resuspended, and stained with 1 µg each of FITC-CD3, BV605-CD4, Alexa Fluor700-CD8, APC-CD25, and PerCP-Cy5.5-CD69 antibodies for 20 min at room temperature in the dark. After washing, the cells were resuspended and analyzed by flow cytometry. Immunomagnetic bead separation was performed in accordance with the manufacturer’s instructions to isolate CD4^+^T cells and CD8^+^T cells from mouse spleen cells. The proportions of CD25⁺ and CD69⁺ within these populations were then analyzed as described above.

### Analysis of spleen cell proliferation by CFSE staining

The spleen cell suspension was labeled with 5 µM carboxyfluorescein succinimidyl ester (CFSE) and incubated at 37 °C for 15–30 min. After washing with PBS, the cells were plated at a density of 2 × 10^6^ cells/mL in 24-well plates, with three wells per mouse designated for each incubation time point (0, 24, and 72 h). For the 24-hour and 72-hour wells, Dynabeads Mouse T-Activator CD3/CD28 and IL-2 (10 ng/mL) were added to stimulate T-cell activation. At each respective time point (0, 24, and 72 h), the cells were harvested, stained with propidium iodide (PI), and analyzed by flow cytometry.

### Analysis of spleen cell proliferation by IHC

The spleen tissue from the mouse was fixed with 4% paraformaldehyde and embedded in paraffin. Tissue sections of 4 μm thickness were then deparaffinized and rehydrated. Primary antibodies Ki67 (1: 600) and PCNA (1: 10000) were diluted and added to the spleen tissue sections. After overnight incubation at 4 °C, a biotinylated secondary antibody was added for 1 h at room temperature. Streptavidin-peroxidase was then added and incubated for 10 min at room temperature. The sections were stained with 3,3’-diaminobenzidine (DAB) and hematoxylin, mounted with neutral resin, and imaged at 400× magnification.

### Analysis of expression levels of CDKs/Cyclins pathway and MAPK pathway related proteins by Western blot

Collected bone marrow cells were used to extract proteins, and protein concentration was determined using the BCA technique. After 10% SDS-PAGE, the lysate’s supernatant, which contained around 50 µg of protein, was transferred onto a polyvinylidene fluoride (PVDF) membrane. Primary antibodies were used to assay the membranes for CDK2 (1:500), CDK4 (1:1000), CDK6 (1:1000), CyclinD1 (1:5000), CyclinE1 (1:500), Rb (1:1000), p-Rb (1:1000), c-Fos (1:1000), p-c-Fos (1:1000), c-Jun (diluted 1:1000), p-c-Jun (1:1000), ERK (1:1000), p-ERK (1:2000), NF-κB p65 (1:1000), p-NF-κB p65 (1:1000), p38 ( 1:1000), p-p38 (1:1000), GAPDH (1:5000), and β-actin (1:1000). Afterwards, horseradish peroxidase (HRP)-conjugated secondary antibodies (1:10000) were incubated. For chemiluminescence, film exposure, and fixation, Millipore ECL (Millipore, MA, USA) was utilized. Image Lab software (BioRad, Hercules, CA, USA) was used to evaluate the grayscale values.

### Statistical analysis

The data obtained from this study were plotted using Prism Graphpad 10.0 and processed and analyzed using SPSS 25.0 statistical software. Survival rates were compared through intergroup survival analysis, while continuous measurement indicators were analyzed using repeated measures ANOVA. For multiple group comparisons, one-way ANOVA was performed, followed by LSD-*t* test when variance was homogeneous or Games-Howell test when variance was heterogeneous, with parameter values expressed as mean ± standard deviation $(\overline{x} \pm s)$. A *P*-value < 0.05 was considered statistically significant.

## Results

### BBD chemical constituents identified by non-targeted metabolomics

**Fig. 1:**
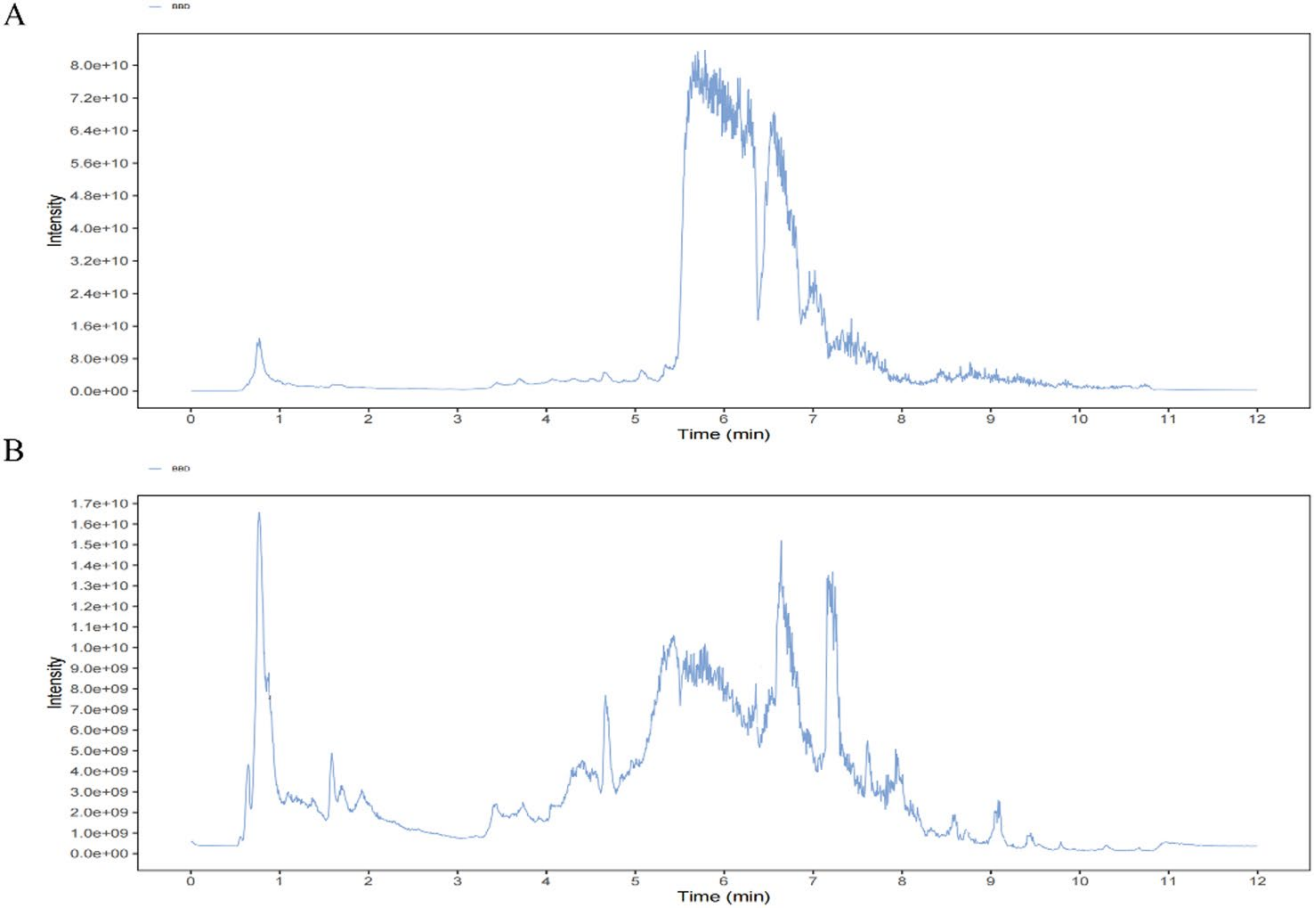
LC-MS/MS analysis of the compounds in BBD. (**A**) The positive ion mode total ion flow diagram. (**B**) The negative ion mode total ion flow diagram.

The chemical constituents of BBD were initially characterized using non-targeted metabolomics. The positive and negative ion mode total ion chromatograms for compound identification in the powder are presented (Fig. 1A and B). A total of 1755 compounds in BBD matched entries in the BiotreeDB (V3.0) database. The major identified components included fatty acids, triterpenoids, flavonoids, alkaloids, and related compounds (Table 1).

**Table 1:** Chemical components of BBD particle.

Rank	Name	Molecular formula	m/z	RT (time)	Area
1	Crotonoside	C10H13N5O5	284.099	53.3	22641081.34
2	Undecanoic acid	C11H22O2	185.1545	492.2	13225424.77
3	Vanillin	C8H8O3	151.04	276.1	65781917.86
4	isofraxidin	C11H10O5	221.0454	280.1	1,329,621,858
5	Diosgenin	C27H42O3	415.3204	485.4	26772538.36
6	Ginsenoside Rb1	C54H92O23	1107.5941	383.7	41526276.64
7	Leucoside	C26H28O15	581.1503	298.2	28336998.43
8	5,7-Dihydroxy-2-(4-hydroxyphenyl)-6-[3,4,5-trihydroxy-6-(hydroxymethyl)tetrahydropyran-2-yl]chroman-4-one	C21H22O10	433.1139	276.1	15205292.13
9	Leucoside	C26H28O15	581.1503	298.2	28336998.43
10	Theophylline	C7H8N4O2	179.0561	195.6	8274150.043
11	1,7-Dimethylxanthine	C7H8N4O2	179.0561	195.6	8274150.043
12	Ginsenoside Rf	C42H72O14	801.4984	332.2	28359148.36
13	7-Methylxanthine	C6H6N4O2	165.0403	65.7	13324931.09
14	Ginsenoside Rg6	C42H70O12	749.4827	412.6	20799210.42
15	Myristic acid	C14H28O2	227.2015	539.4	67135264.7
16	Alpha-Linolenic acid	C18H30O2	277.217	531	67946174.63
17	1,7-Diazatricyclo[7.3.0.03,7]dodecane-2,8-dione	C10H14N2O2	195.1128	211.7	33448516.38
18	Ginsenoside Re	C48H82O18	945.5421	412.3	25595565.21
19	7-Ketolithocholic acid	C24H38O4	373.2731	431.1	3,299,494,186
20	6-Hydroxyluteolin 7-glucoside	C21H20O12	465.1022	284	10685983.61
21	Cubebinin	C24H32O8	449.2162	280.7	14063558.99
22	Benzyl beta-d-glucopyranoside	C13H18O6	288.1439	249.3	8162402.477
23	Alpha-Asarone	C12H16O3	209.117	270.4	18477581.07
24	3,4-dimethyl-2,5-bis(3,4,5-trimethoxyphenyl)oxolane	C24H32O7	450.245	202.2	11522164.07
25	4-(2,3-Dihydroxy-3-methylbutoxy)furo(3,2-g)chromen-7-one	C16H16O6	303.0832	88.4	8288347.865
26	5-Methoxypsoralen	C12H8O4	215.0331	656.9	13425180.03
27	7-[(2,6-dihydroxy-2,5,5,8a-tetramethyl-3,4,4a,6,7,8-hexahydro-1 H-naphthalen-1-yl)methoxy]chromen-2-one	C24H32O5	401.2319	451.4	25690840.04
28	(-)^®^2-(4-hydroxy-2-oxoindolin-3-yl)acetonitrile	C10H8N2O2	189.0657	177.6	8267901.06
29	3-hydroxyphenylglycine	C8H9NO3	168.0653	150.6	7419281.752
30	5-O-Methylembelin	C18H28O4	307.1911	462.8	12651862.1

### BBD mitigates the decreased survival rate, weight loss, and spleen atrophy induced by 5-FU

**Fig. 2:**
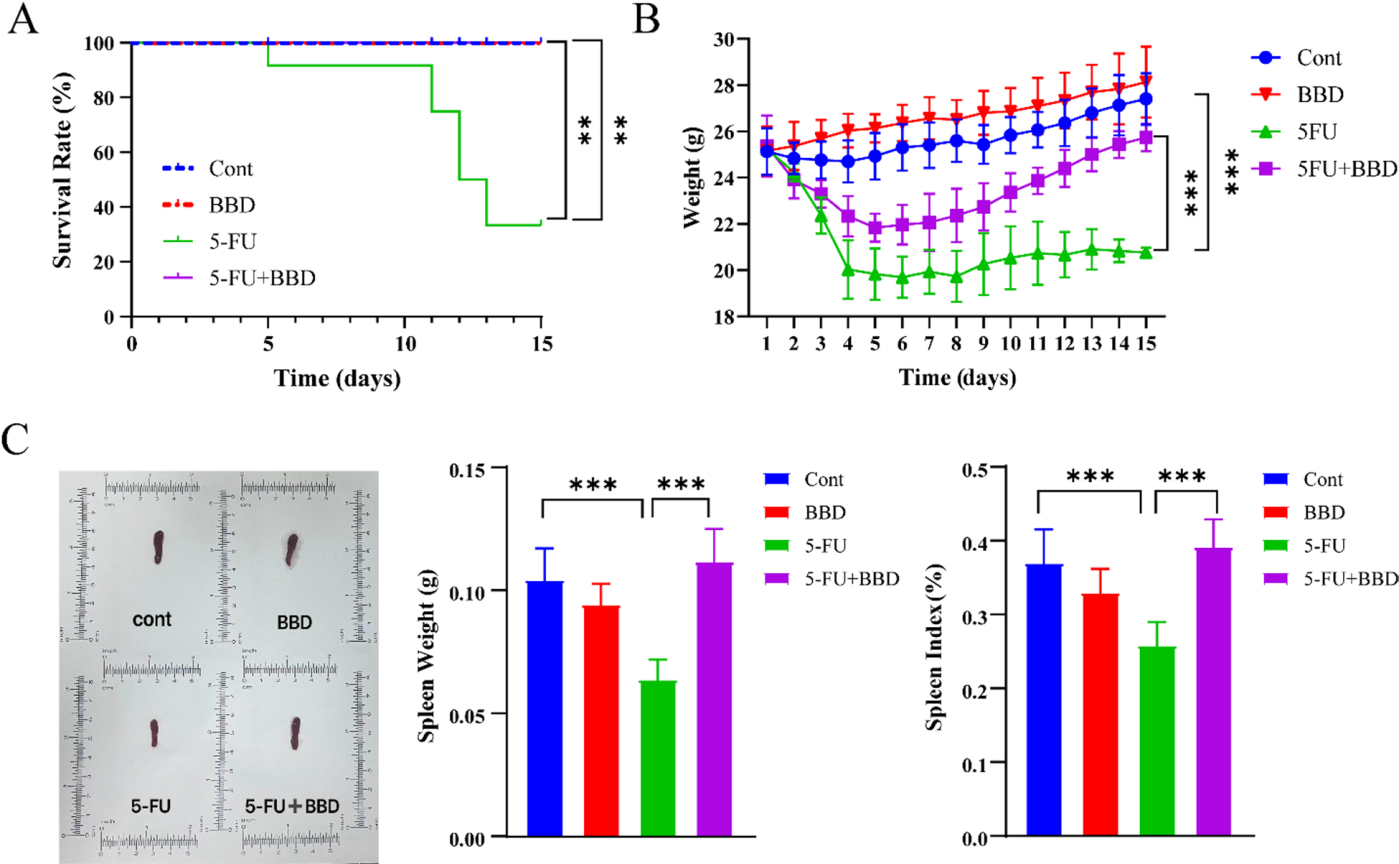
BBD mitigates the decreased survival rate, weight loss, and spleen atrophy induced by 5-FU. (**A**) Survival rate. (**B**) Body weight. (**C**) Spleen size, Spleen weight, and spleen index. Data were shown as mean ± S.D. **P < 0.01, and ***P < 0.001.

To investigate whether BBD can improve 5-FU-induced mortality, we used mice treated with a dosage of 50 mg/kg of 5-FU. A significant mortality rate was observed in comparison to the Cont group, with a survival rate of only 33.3% after 15 days (*P* < 0.01). In contrast, the 5-FU + BBD group exhibited markedly improved survival outcomes, with no recorded fatalities (*P* < 0.01, Fig. 2A). At a dosage of 30 mg/kg of 5-FU, the 5-FU group exhibited a significant reduction in body weight compared to the Cont group (*P* < 0.001). In contrast, the 5-FU + BBD group exhibited a significant improvement in body weight, suggesting that BBD could alleviate the loss of body weight induced by 5-FU (*P* < 0.001, Fig. 2B).

As a pivotal peripheral immune organ, variations in splenic size and organ index offer valuable insights into the functional state of the immune system. Our results showed that the 5-FU group exhibited a reduction in spleen size, with a significant decrease in spleen weight (*P* < 0.001) and spleen index (*P* < 0.001) compared to the Cont group. In contrast, the 5-FU + BBD group showed an increase in both spleen weight and spleen index compared to the 5-FU group (*P* < 0.001, Fig. 2C). These findings suggest that BBD can effectively mitigate the immune damage induced by 5-FU.

### BBD mitigates the inhibitory effects of 5-FU on bone marrow hematopoiesis

**Fig. 3:**
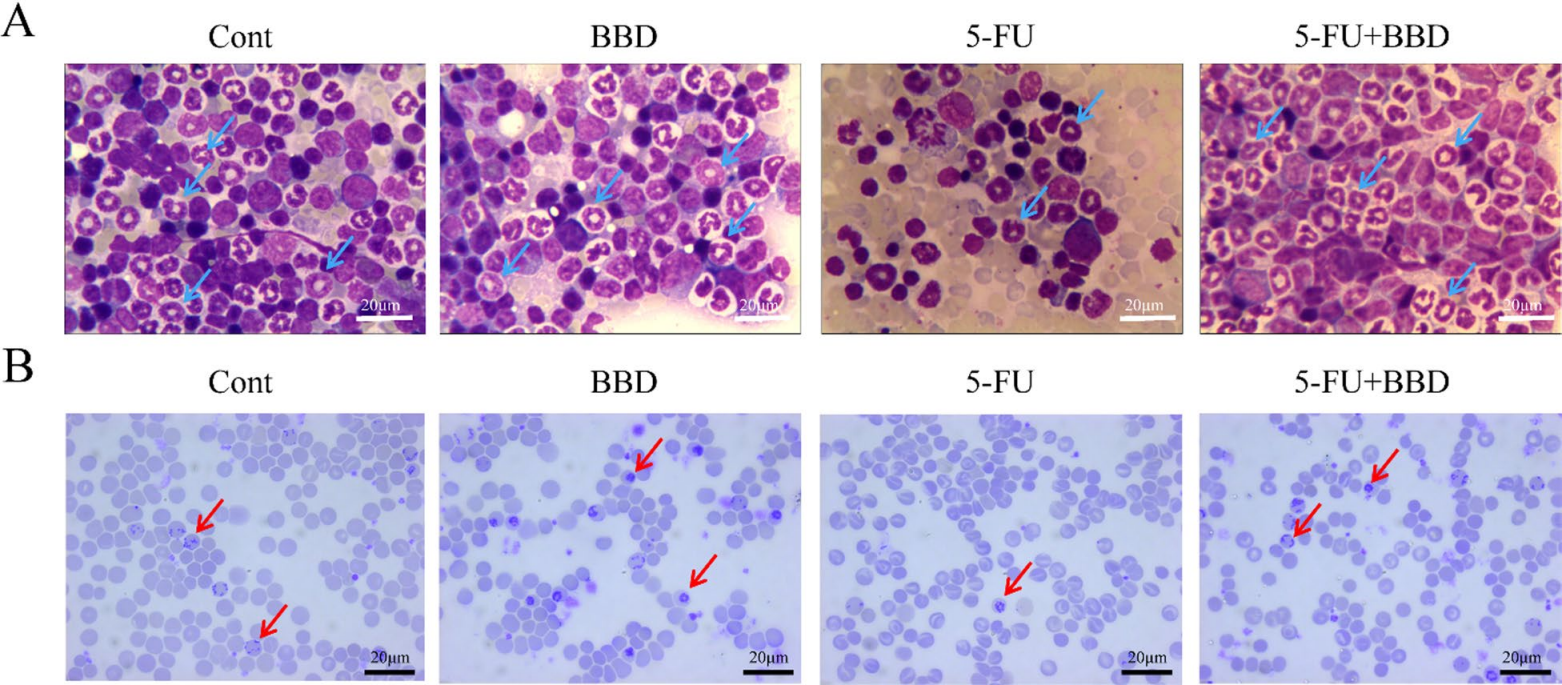
BBD alleviates 5-FU-induced suppression of bone marrow hematopoietic function. (**A**) Mice bone marrow cells stained with Wright-Giehl method, arrows indicate band/segmented neutrophils, scale bars: 20 μm. (**B**) Mice peripheral blood reticulocytes (arrowed) stained with Brilliant Tar Blue method, scale bars: 20 μm.

To elucidate the impact of BBD on 5-FU-induced myelosuppression, we conducted observations on myeloid and lymphoid cells in the bone marrow as well as reticulocytes in peripheral blood. As depicted in the representative images (Fig. 3A) and tables of bone marrow cellularity (Tables 2, 3 and 4), the comparison of erythrocyte levels across mouse groups showed no statistically significant differences (Table 2). In contrast, the 5-FU group exhibited a significant reduction in mature granulocytes, such as band and segmented neutrophils (*P* < 0.05, Table 3), while the proportion of mature monocytes was markedly increased (*P* < 0.05, Table 4). Compared with the 5-FU group, the 5-FU + BBD group demonstrated a substantial increase in mature granulocytes, including band and segmented neutrophils (*P* < 0.001, Table 3). These findings indicate that 5-FU inhibits the differentiation and maturation of granulocytes in mice, leading to a relative increase in the proportions of monocytes. BBD, however, promotes the differentiation and maturation of granulocytes in mice.

**Table 2:** Effects of BBD on erythrocytes in mouse bone marrow $(\overline {x} \pm s)$.

Group	Proerythroblasts (%)	Basophilic erythroblasts (%)	Polychromatic erythroblasts (%)	Orthochromatic erythroblast (%)
Cont	0.5 ± 0.71	2.00 ± 1.96	18.63 ± 5.41	10.5 ± 2.94
BBD	0.4 ± 0.54	1.13 ± 0.25	18.35 ± 3.93	10.63 ± 1.60
5-FU	0.67 ± 0.74	0.83 ± 0.76	12.67 ± 13.61	6.50 ± 5.77
5-FU + BBD	0.53 ± 0.26	1.17 ± 0.29	2.30 ± 1.53	0.50 ± 0.50

**Table 3:** Effects of BBD on granulocytes in mouse bone marrow $(\overline {x} \pm s)$.

Group	Myelocytes (%)	Metamyelocytes (%)	Band/Segmented Neutrophils (%)
Cont	1.00 ± 0.71	3.88 ± 1.80	39.4 ± 3.05
BBD	1.38 ± 0.75	7.38 ± 3.01	42.75 ± 1.32
5-FU	2.1 ± 0.29	10.6 ± 2.70	25.13 ± 3.14*
5-FU + BBD	0.50 ± 0.5	7.83 ± 2.75	77.00 ± 6.76^###^

*P<0.05, compared with the Cont group; ^###^*P*<0.001, compared with the 5-FU group.

**Table 4:** Effects of BBD on lymphocytes and monocytes in mouse bone marrow $(\overline {x} \pm s)$.

Group	Mature lymphocytes (%)	Mature monocytes (%)
Cont	22.75 ± 6.85	0.25 ± 0.29
BBD	17.00 ± 5.15	0.25 ± 0.29
5-FU	39.83 ± 35.25	1.67 ± 1.04*
5-FU + BBD	7.00 ± 3.77	1.83 ± 2.31

*P<0.05, compared with the Cont group.

Reticulocytes were observed to be dispersed among blood cells, exhibiting a blue, punctate, or reticular cell structure following staining with BCB. In the Cont and BBD groups, 5–7 reticulocytes per field of view were consistently observed, whereas in the 5-FU group, 0–1 reticulocyte per field of view was occasionally detected. In comparison with the 5-FU group, the number of reticulocytes in the 5-FU + BBD group exhibited an increasing trend, with 3–5 reticulocytes per field of view being observed (Fig. 3B). Quantitative analysis of reticulocyte ratios and maturation levels was performed using a multi-parameter hematology analyzer. Compared with the Cont group, the 5-FU group exhibited significant reductions in reticulocyte absolute count, reticulocyte percentage, medium-fluorescence reticulocyte ratio, high-fluorescence reticulocyte ratio, and immature reticulocyte ratio (*P* < 0.05 or *P* < 0.001). Conversely, the low-fluorescence reticulocyte ratio was significantly elevated (*P* < 0.01). In comparison with the 5-FU group, the 5-FU + BBD group demonstrated an increased reticulocyte absolute count and decreased ratios of medium-fluorescence reticulocytes, high-fluorescence reticulocytes, and immature reticulocytes (*P* < 0.05), while the low-fluorescence reticulocyte ratio was elevated (*P* < 0.05, Table 5). These findings suggest that 5-FU impairs bone marrow hematopoietic function in mice, whereas BBD attenuates the decline in bone marrow hematopoietic function following 5-FU chemotherapy.

**Table 5:** Effects of BBD on the number and ratio of reticulocytes in mice $(\overline {x} \pm s)$.

Group	RET# (10^9^/L)	RET (%)	LFR (%)	MFR (%)	HFR (%)	IRF (%)
Cont	294.70 ± 22.72	2.94 ± 0.28	58.13 ± 1.95	17.37 ± 3.19	24.5 ± 1.45	41.87 ± 1.95
BBD	306.40 ± 31.81	3.08 ± 0.15	58.83 ± 4.35	16.87 ± 0.83	24.3 ± 3.80	41.17 ± 4.35
5-FU	110.80 ± 7.71***	1.30 ± 0.23	87.30 ± 12.38**	6.67 ± 4.09*	6.03 ± 9.00*	12.71 ± 2.38^*^
5-FU + BBD	881.50 ± 511.16^#^	9.63 ± 5.11	63.77 ± 2.91^#^	18.8 ± 5.99^#^	17.43 ± 3.54	36.23 ± 2.91^#^

*P<0.05, **P<0.01 and ***P<0.001, compared with the Cont group; ^#^*P*<0.05, compared with the 5-FU group.

RET#: reticulocyte absolute value; RET: reticulocyte; LFR: percentage of low fluorescence intensity reticulocytes; MFR: percentage of medium fluorescence intensity reticulocytes; HFR: percentage of high fluorescence intensity reticulocytes; IRF: percentage of immature reticulocytes.

Peripheral blood analysis revealed that the mice in the 5-FU group showed a significant decrease in leukocyte count, absolute neutrophil count, and absolute lymphocyte count (*P* < 0.05, Table 6), as well as haemoglobin concentration (*P* < 0.05, Table 7) compared with the Cont group, along with a rise in platelet count (*P* < 0.05, Table 8). Conversely, the mice in the 5-FU + BBD group demonstrated a significant increase in leukocyte count, absolute neutrophil count, absolute lymphocyte count (*P* < 0.05, Table 6), and platelet count relative to the 5-FU group (*P* < 0.05, Table 8). The results indicated that 5-FU chemotherapy inhibited the production of both leukocytes and haemoglobin in the blood of mice, with a more pronounced suppression observed in the leukocytes, while BBD could significantly ameliorate the inhibition of leukocyte count induced by 5-FU.

**Table 6:** Effects of BBD on blood routine indexes of mice in each group $(\overline {x} \pm s)$.

Group	WBC (10^9^/L)	NEUT# (10^9^/L)	LYM# (10^9^/L)	MONO# (10^9^/L)	NEUT (%)	LYM(%)	MONO(%)
Cont	5.15 ± 1.42	1.23 ± 0.18	3.83 ± 1.53	0.05 ± 0.02	26.10 ± 12.23	72.27 ± 11.68	0.97 ± 0.06
BBD	4.23 ± 1.40	0.97 ± 0.21	3.19 ± 1.27	0.06 ± 0.03	23.87 ± 5.58	74.40 ± 5.65	1.37 ± 0.25
5-FU	1.28 ± 0.58*	0.31 ± 0.39*	0.6 ± 10.14*	0.36 ± 0.62	26.53 ± 25.56	52.77 ± 17.06	20.70 ± 35.85
5-FU + BBD	6.21 ± 2.15^#^	2.44 ± 0.8^#^	3.05 ± 0.54^#^	0.71 ± 0.95	39.40 ± 1.15	51.30 ± 9.80	9.171 ± 0.55

*P<0.05, compared with the Cont group; ^#^*P*<0.05, compared with the 5-FU group.

WBC: white blood cell count; NEUT#: absolute neutrophil count; LYM#; absolute lymphocyte count; MONO#: absolute monocyte count; NEUT: the percent of neutrophil; LYM: the percent of lymphocyte; MONO: the percent of monocyte.

**Table 7:** Effects of BBD on various parameters related to mouse erythrocytes $(\overline {x} \pm s)$.

Group	RBC (10^9^/L)	HGB (g/L)	HCT(%)	RDW(%)
Cont	10.05 ± 0.30	150 ± 7.94	16.63 ± 0.74	14.87 ± 0.38
BBD	9.94 ± 0.62	149 ± 7.55	17.00 ± 1.31	15.03 ± 0.49
5-FU	8.66 ± 1.22	129 ± 21.00*	14.23 ± 1.36	15.43 ± 1.00
5-FU + BBD	8.84 ± 0.96	133 ± 16.52	15.43 ± 1.72	18.37 ± 0.67^#^

*P<0.05, compared with Cont group; ^#^*P*<0.05, compared with 5-FU group.

RBC: red blood cell count; HGB: hemoglobin; HCT: hematocrit; RDW: red cell volume distribution width.

**Table 8:** Effects of BBD on platelet-related indicators of mice in each group $(\overline {x} \pm s)$.

Group	PLT (10^9^/L)	MPV (fL)	PCT (%)	PDW (%)
Cont	1058.00 ± 155.37	7.83 ± 0.15	0.15 ± 0.02	9.70 ± 0.44
BBD	916.00 ± 85.35	8.03 ± 0.47	0.17 ± 0.04	10.57 ± 1.01
5-FU	1954.00 ± 538.81*	7.90 ± 0.35	0.29 ± 0.17	9.23 ± 0.81
5-FU + BBD	3012.00 ± 471.72^#^	8.37 ± 0.31	0.54 ± 0.05	11.3 ± 1.15

*P<0.05, compared with the Cont group; ^#^*P*<0.05, compared with the 5-FU group.

PLT: platelet count; MPV: mean platelet volume; PCT: plateletcrit; PDW: platelet distribution width.

Our findings demonstrate that BBD effectively alleviates 5-FU-induced hematopoietic suppression through promoting granulocyte differentiation and maturation, restoring reticulocyte production, and reversing chemotherapy-associated leukopenia. These results establish BBD’s myeloprotective capacity against 5-FU toxicity, suggesting its potential as an adjunctive therapy for chemotherapy-induced myelosuppression.

### BBD alleviates 5-FU-induced leukopenia and enhances NK cell proportion in Splenic leukocytes

**Fig. 4:**
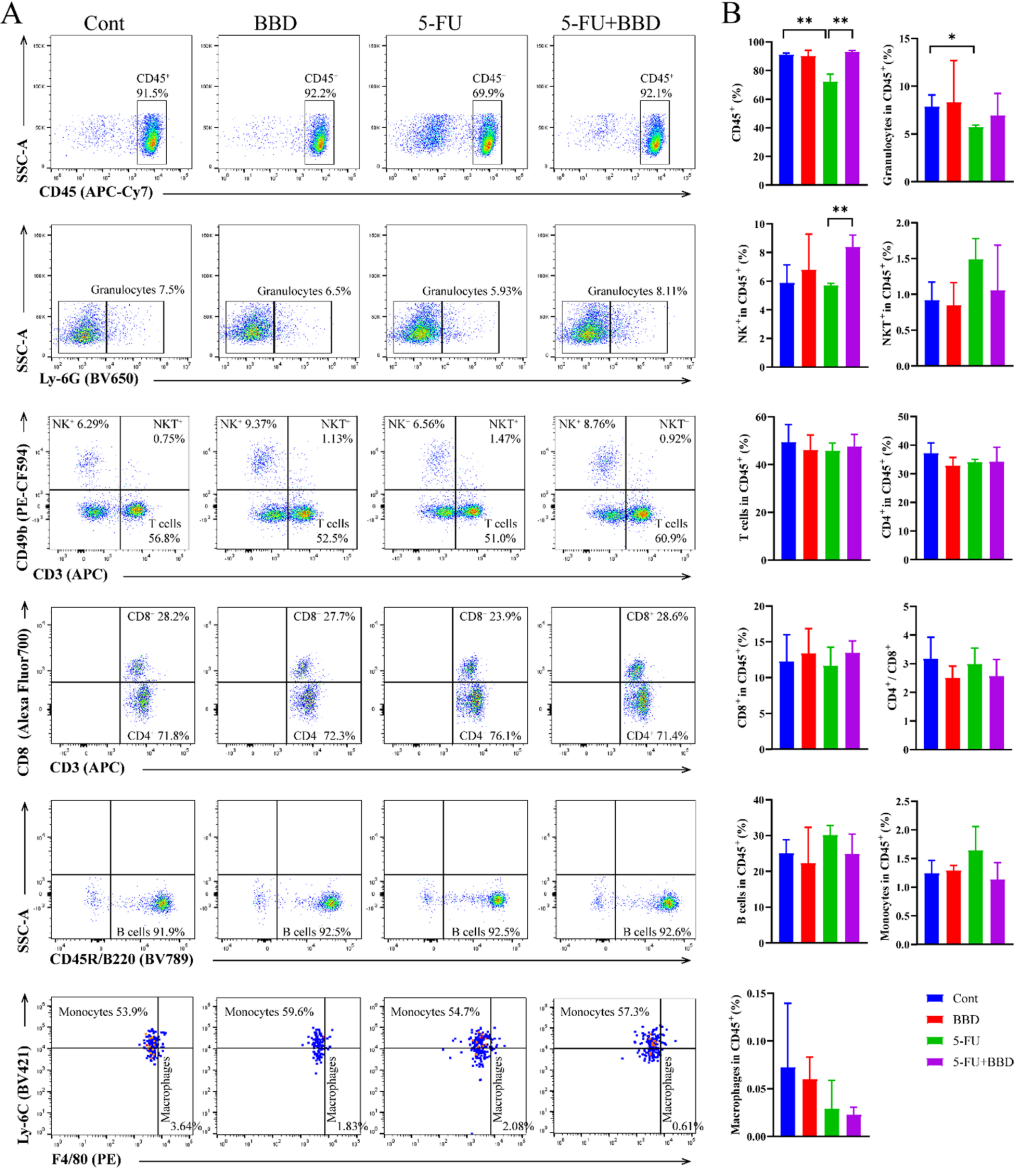
BBD alleviates 5-FU-induced leukopenia and increases the proportion of NK cells in splenic leukocytes. (**A**) Flow cytometry plots showing cell subpopulations in mouse spleens: CD45⁺ cells, granulocytes, NK cells, NKT cells, T cells, CD4⁺T cells, CD8⁺T cells, B cells, monocytes, and macrophages. (**B**) The proportion of CD45⁺ cells, granulocytes, NK cells, NKT cells, T cells, CD4⁺T cells, CD8⁺T cells, B cells, monocytes, and macrophages in mouse spleens. Data were shown as mean ± S.D. *P < 0.05, and **P < 0.01.

To examine the potential of BBD in ameliorating immune disorders induced by 5-FU, we analyzed the distribution of leukocytes within the spleen, a key peripheral immune organ. A significant decrease in leukocyte (CD45^+^) levels was observed following 5-FU chemotherapy, with granulocytes being predominantly affected. No statistically significant alterations were detected in other leukocyte subpopulations. Importantly, BBD was found to effectively mitigate this chemotherapy-induced leukopenia while concurrently elevating the percentage of NK cells (Fig. 4A-B).

### BBD modulates the CD4^+^/CD8^+^T cell balance in 5-FU-treated mice

As previously shown, 5-FU did not significantly alter T cell subpopulation proportions in splenocytes. Therefore, we examined T cell subpopulation changes in peripheral blood across different groups of mice to determine whether these changes paralleled those in the spleen.

**Fig. 5:**
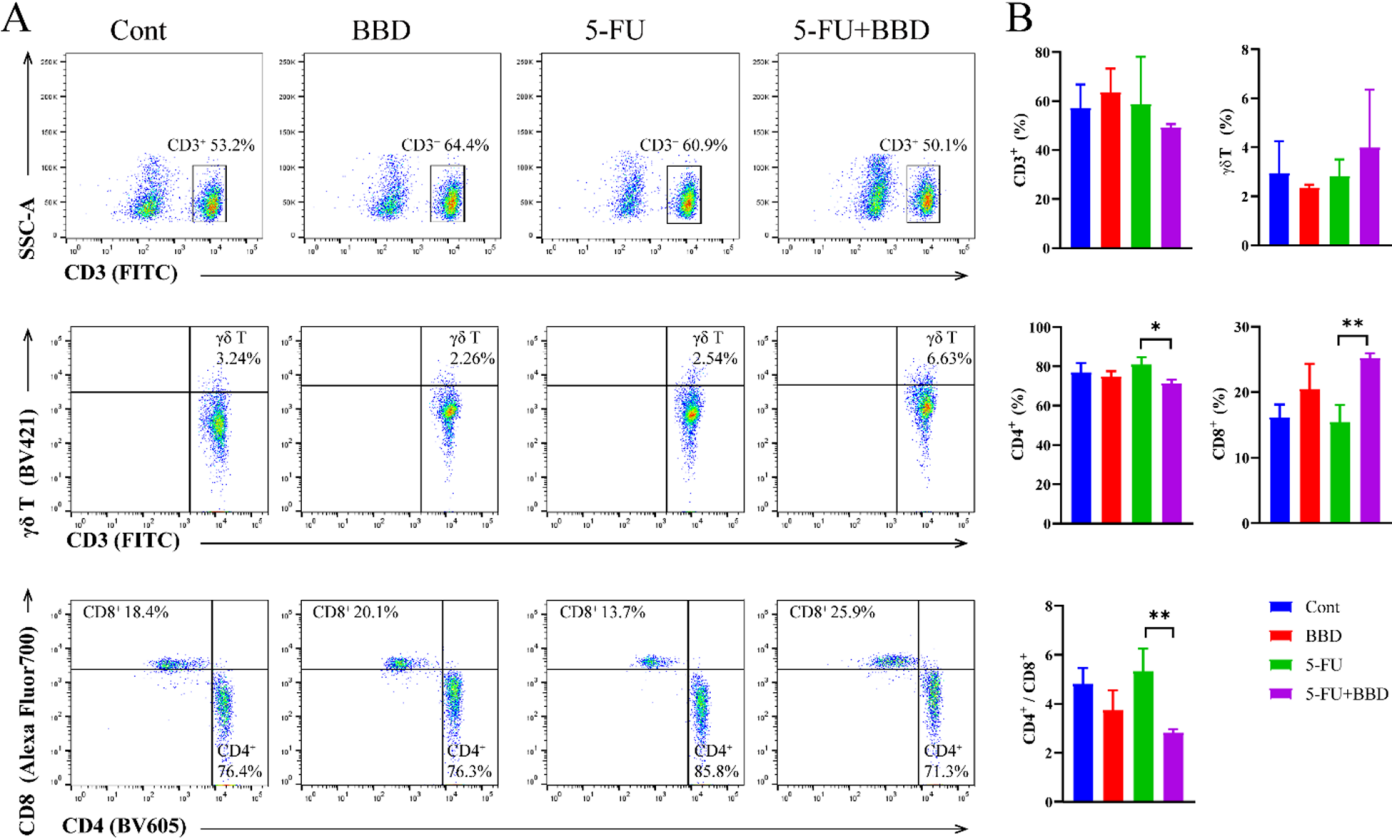
BBD modulates the CD4^+^/CD8^+^T cell balance in 5-FU-treated mice. (**A**) Flow cytometry plots showing cell subpopulations in mouse peripheral blood: CD3^+^T cells, γδT cells, CD4^+^T cells, CD8^+^T cells. (**B**) The proportion of CD3^+^T cells, γδT cells, CD4^+^T cells, CD8^+^T cells and the ratio of CD4^+^T cells to CD8^+^T cells in mouse peripheral blood. Data were shown as mean ± S.D. *P < 0.05, and **P < 0.01.

Compared with the Cont group, 5-FU treatment did not significantly affect T cell subpopulation proportions in mice. In contrast, the 5-FU + BBD group exhibited a significant decrease in CD4^+^T cells (*P* < 0.05) and an increase in CD8^+^T cells (*P* < 0.01), resulting in a reduced CD4^+^/CD8^+^ ratio (*P* < 0.01) (Fig. 5A-B). These results indicate that 5-FU chemotherapy has minimal impact on peripheral blood T cell subpopulations, similar to observations in splenocytes. However, BBD modulates the balance between CD4^+^T and CD8^+^T cells in the peripheral blood of 5-FU-treated mice.

### BBD modulates Th1/Treg balance and protects terminally differentiated effector memory cells post-5-FU chemotherapy

**Fig. 6:**
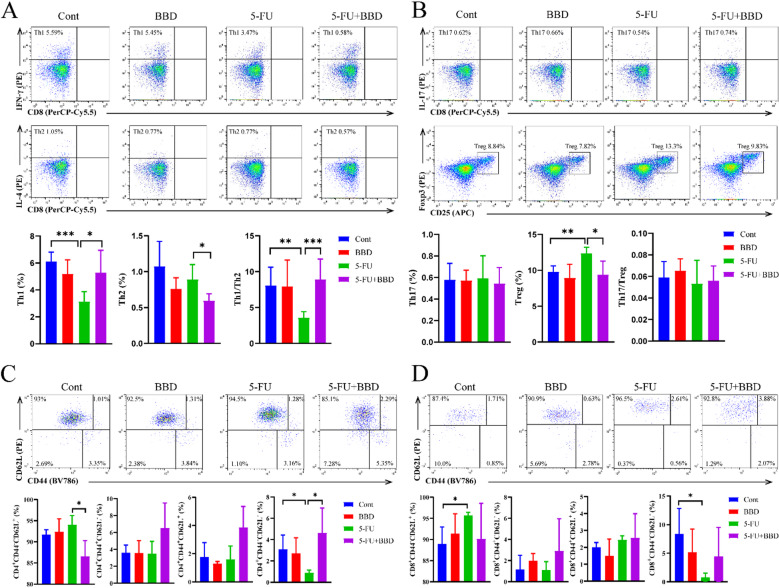
BBD ameliorates 5-FU-induced Th1/Th2 imbalance and reduces 5-FU killing of terminally differentiated effector memory cells. (**A**) The proportion of Th1, Th2 cell subsets of CD4^+^T cells in the spleens of mice, and analysis of the ratio of Th1 and Th2. (**B**) The proportion of Th17, Treg cell subsets of CD4^+^T cells in the spleens of mice, and analysis of the ratio of Th17 and Treg. (**C**) The proportion of naïve T cells, effector memory T cells, central memory T cells and terminally differentiated effector memory cells subpopulations among CD4^+^T cells in peripheral blood of mice. (**D**) The proportion of naïve T cells, effector memory T cells, central memory T cells and terminally differentiated effector memory cells subpopulations among CD8^+^T cells in peripheral blood of mice. Data were shown as mean ± S.D. *P < 0.05, **P < 0.01, and ***P < 0.001.

To further explore the impact of 5-FU on the distribution of CD4^+^T cell subsets within splenic leukocytes, as well as the potential influence of BBD, we analyzed the proportions of Th1, Th2, Th17, and Treg cells in the spleen. Compared with the Cont group, the 5-FU group showed significantly reduced Th1 proportions (*P* < 0.001) and Th1/Th2 ratios (*P* < 0.01, Fig. 6A), accompanied by a significant elevation in Treg proportions (*P* < 0.01, Fig. 6B). Conversely, the 5-FU + BBD group exhibited higher Th1 proportions (*P* < 0.05), lower Th2 and Treg proportions (*P* < 0.05), along with normalization of Th1/Th2 homeostasis (*P* < 0.001, Fig. 6A-B). These results suggest that 5-FU administration induces immunosuppressive effects through Th1/Th2 axis imbalance and Treg potentiation, while BBD counteracts these immunological perturbations through coordinated regulation of Th1/Th2 balance and Treg proportions.

To further investigate the impact of BBD on peripheral T cell immune responses post-5-FU chemotherapy, we analyzed the proportions of naïve T cells (CD44^−^CD62L^+^), effector memory T cells (CD44^+^CD62L^−^), central memory T cells (CD44^+^CD62L^+^) and terminally differentiated effector memory cells (CD44^−^CD62L^−^). The 5-FU group exhibited increased naïve T cell proportions within CD8^+^T cells (*P* < 0.05) compared with the Cont group, while a reduction in terminally differentiated effector memory cell proportions was observed in both CD4^+^T cells and CD8^+^T cells (*P* < 0.05, Fig. 6C-D). In contrast, the 5-FU + BBD group showed decreased naïve T cell proportions within CD4^+^T cells (*P* < 0.05), accompanied by an increase in terminally differentiated effector memory cell proportions specifically in CD4^+^T cells compared to the 5-FU group (*P* < 0.05, Fig. 6C). These findings indicate that 5-FU preferentially targets terminally differentiated effector memory cells, with minimal effects on naïve T cells, leading to a relative increase in naïve T cell proportions. BBD appears to restore the balance among T cell subsets by reducing 5-FU-induced cytotoxicity in terminally differentiated effector memory cells.

### BBD restores T cell activation in splenocytes post-5-FU chemotherapy

**Fig. 7:**
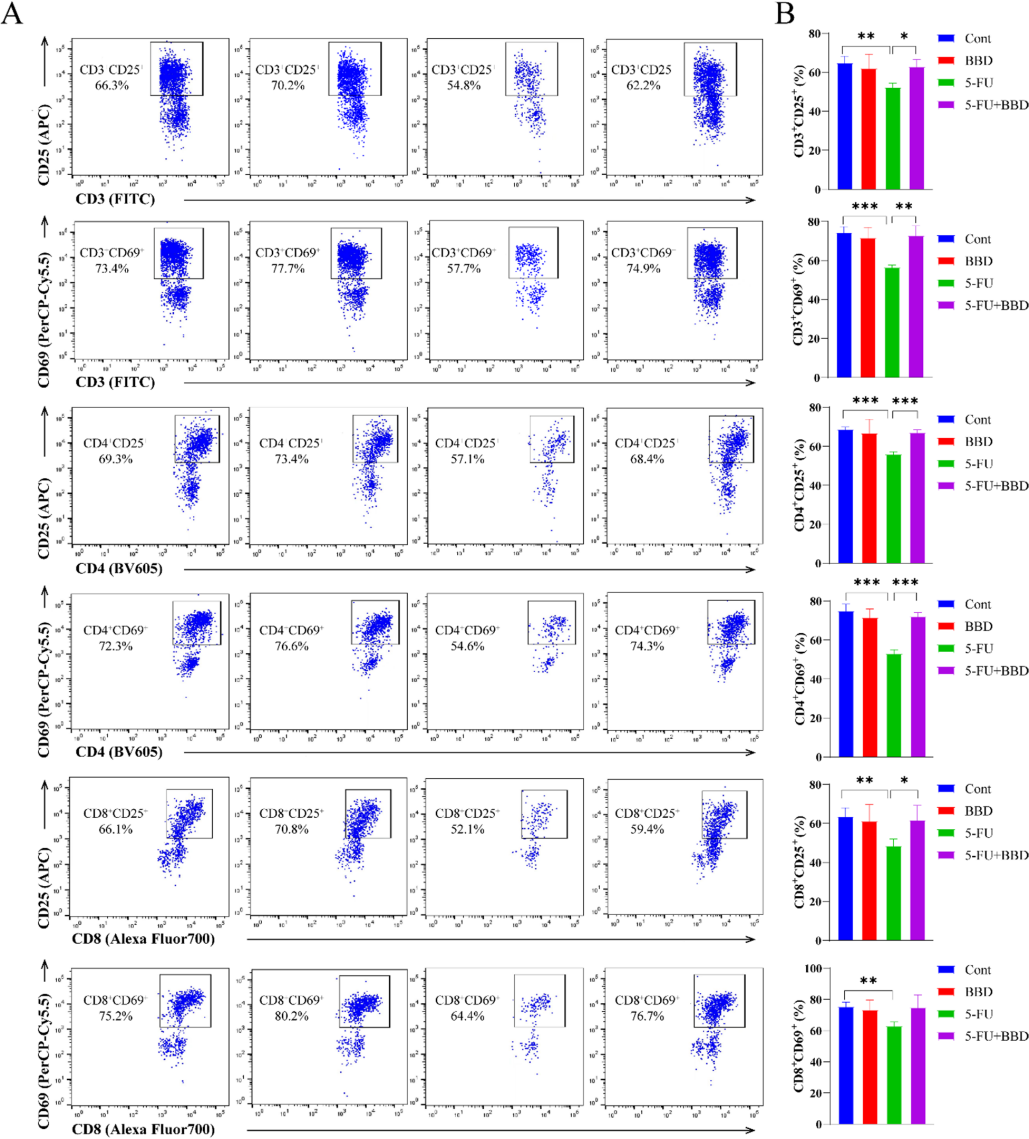
BBD ameliorates 5-FU-induced inhibition of T-cell activation. (**A**) Flow cytometry plots showing cell subpopulations in mouse spleens: CD25 and CD69 in CD3^+^T cells as well as CD4^+^T cells and CD8^+^T cells. (**B**) The proportion of CD25 and CD69 in CD3^+^T cells as well as CD4^+^T cells and CD8^+^T cells in mouse spleens. Data were shown as mean ± S.D. *P < 0.05, **P < 0.01, and ***P < 0.001.

To assess the impact of BBD on T lymphocyte activation post-5-FU chemotherapy, splenocytes from different groups were stimulated and observed after 6 h. CD69 (early-stage) and CD25 (intermediate-stage) constitute sequential activation markers that coordinately regulate T lymphocytes stimulation processes^19,20^. Compared with the Cont group, following stimulation, splenocytes from mice in the 5-FU group exhibited significantly reduced proportions of CD3^+^CD25^+^, CD3^+^CD69^+^, CD4^+^CD25^+^, CD4^+^CD69^+^, CD8^+^CD25^+^, and CD8^+^CD69^+^ cells (*P* < 0.01 or *P* < 0.001). In contrast, compared with the 5-FU group, these parameters were significantly increased in mice treated with 5-FU + BBD (Fig. 7). These findings indicate that 5-FU suppresses the activation of both CD4^+^ T and CD8^+^T lymphocytes, whereas BBD can ameliorate this inhibition.

### BBD restores 5-FU-suppressed CD25 and CD69 expression in purified CD4^+^T cells and CD8^+^T cells

**Fig. 8:**
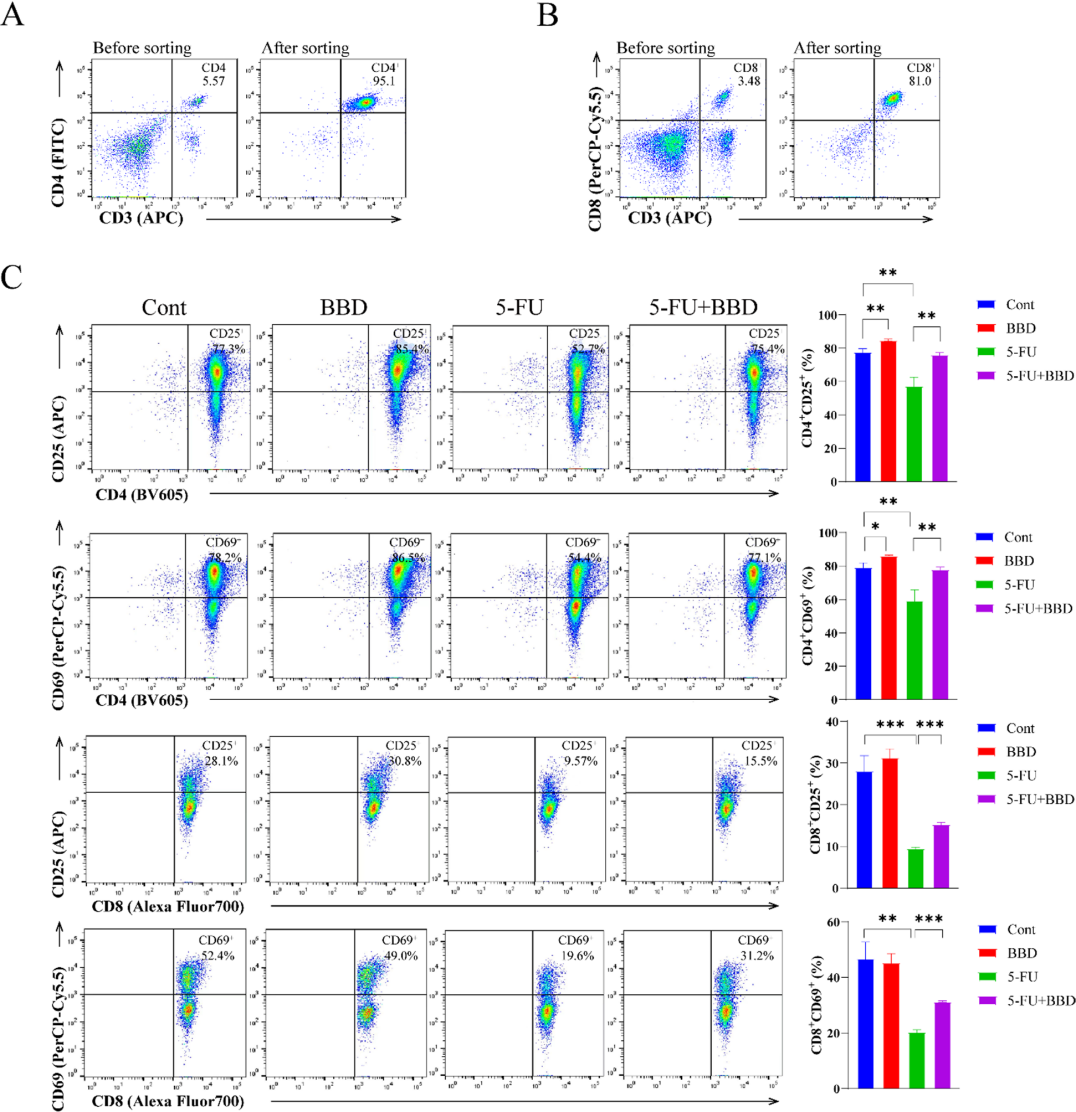
BBD restores the expression of CD25 and CD69 in purified CD4^+^T cells and CD8^+^T cells suppressed by 5-FU. (**A**) Immunomagnetic bead sorting of CD4^+^T cells from mouse splenocytes. (**B**) Immunomagnetic bead sorting of CD8^+^T cells from mouse splenocytes. (**C**) The expression of CD25 and CD69 in splenic CD4^+^T cells and CD8^+^T cells of mice after magnetic beads sorting. Data were shown as mean ± S.D. *P < 0.05, **P < 0.01, and ***P < 0.001.

To further elucidate the activation status of CD25^+^ and CD69^+^ within CD4^+^T cells and CD8^+^T cells in murine splenocytes, we employed magnetic bead sorting to isolate CD4^+^T cells and CD8^+^T cells, followed by the detection of CD25^+^ and CD69^+^ expression. The sorting procedure achieved a purity exceeding 80% (Fig. 8A-B). Compared with the Cont group, mice in the BBD group exhibited increased proportions of CD4^+^CD25^+^ and CD4^+^CD69^+^ cells (*P* < 0.05 or *P* < 0.01). Conversely, the 5-FU group demonstrated significant reductions in the proportions of CD4^+^CD25^+^, CD4^+^CD69^+^, CD8^+^CD25^+^, and CD8^+^CD69^+^ cells (*P* < 0.01 or *P* < 0.001). Compared with the 5-FU group, the 5-FU + BBD group showed elevated proportions of CD4^+^CD25^+^, CD4^+^CD69^+^, CD8^+^CD25^+^, and CD8^+^CD69^+^ cells (*P* < 0.01 or *P* < 0.001) (Fig. 8C). These results further confirm that 5-FU suppresses the activation of both CD4^+^T cells and CD8^+^T cells. BBD not only mitigates this inhibition but also exerts a stimulatory effect on the activation of CD4^+^T cells and CD8^+^T cells in normal mice.

### BBD restores splenocyte proliferation post-5-FU chemotherapy

**Fig. 9:**
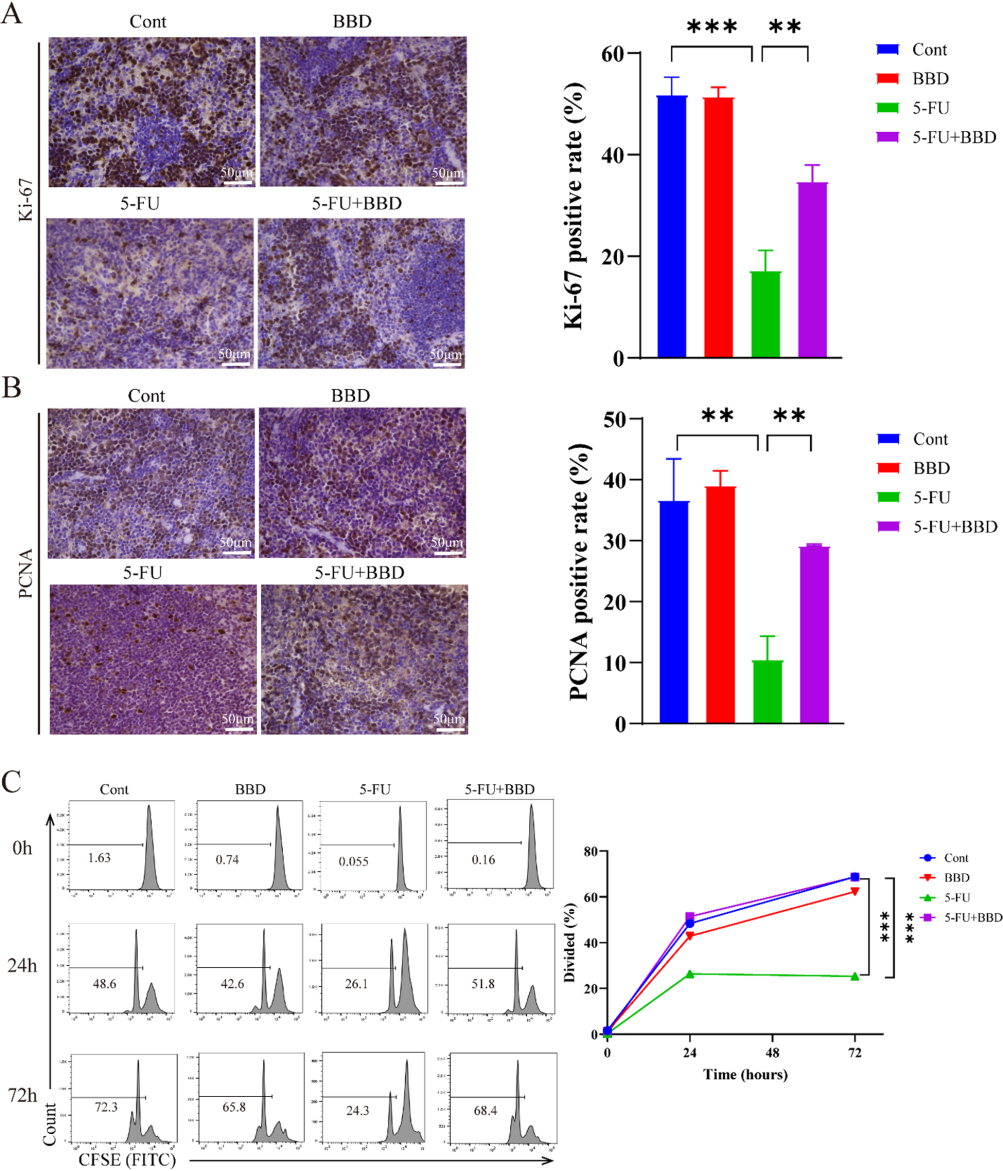
BBD attenuates 5-FU-induced inhibition of cell proliferation in mice. The IHC method was used to stain representative splenic tissue sections from each group to determine the expression of Ki-67 (**A**) and PCNA (**B**), scale bar: 50 μm. CFSE staining to detect the proliferating generations of mice splenocytes at 0 h, 24 h and 72 h (**C**). Data were shown as mean ± S.D. **P < 0.01, and ***P < 0.001.

To assess the impact of BBD on T lymphocyte proliferation post-5-FU chemotherapy, expression of Ki-67 and PCNA, which localize to the cell nucleus and appear as brownish-yellow staining, was assessed. Compared with the Cont group, mice in the 5-FU group exhibited significantly reduced expression of Ki-67 and PCNA within the spleen (*P* < 0.01 or *P* < 0.001). Conversely, in the 5-FU + BBD group, expression of Ki-67 and PCNA were markedly elevated (*P* < 0.01) (Fig. 9A-B). These findings indicate that 5-FU suppresses splenocyte proliferation, whereas BBD mitigates this inhibition and restores proliferative capacity.

In the splenocyte activation assay, the Cont and BBD groups showed progressive increases in cell divisions over time, with 70% of cells proliferating by 72 h. In contrast, the 5-FU group showed significantly suppressed proliferation, with only 25% of cells undergoing division (*P* < 0.001). Notably, the 5-FU + BBD group displayed a marked recovery in proliferative activity, reaching 68% of proliferating cells (*P* < 0.001), a level comparable to the Cont group (Fig. 9C). These results indicate that BBD effectively counteracts 5-FU-induced suppression of splenocyte proliferation.

### BBD mitigates 5-FU-induced myelosuppression by modulating cell cycle progression and MAPK signaling pathway activation

**Fig. 10:**
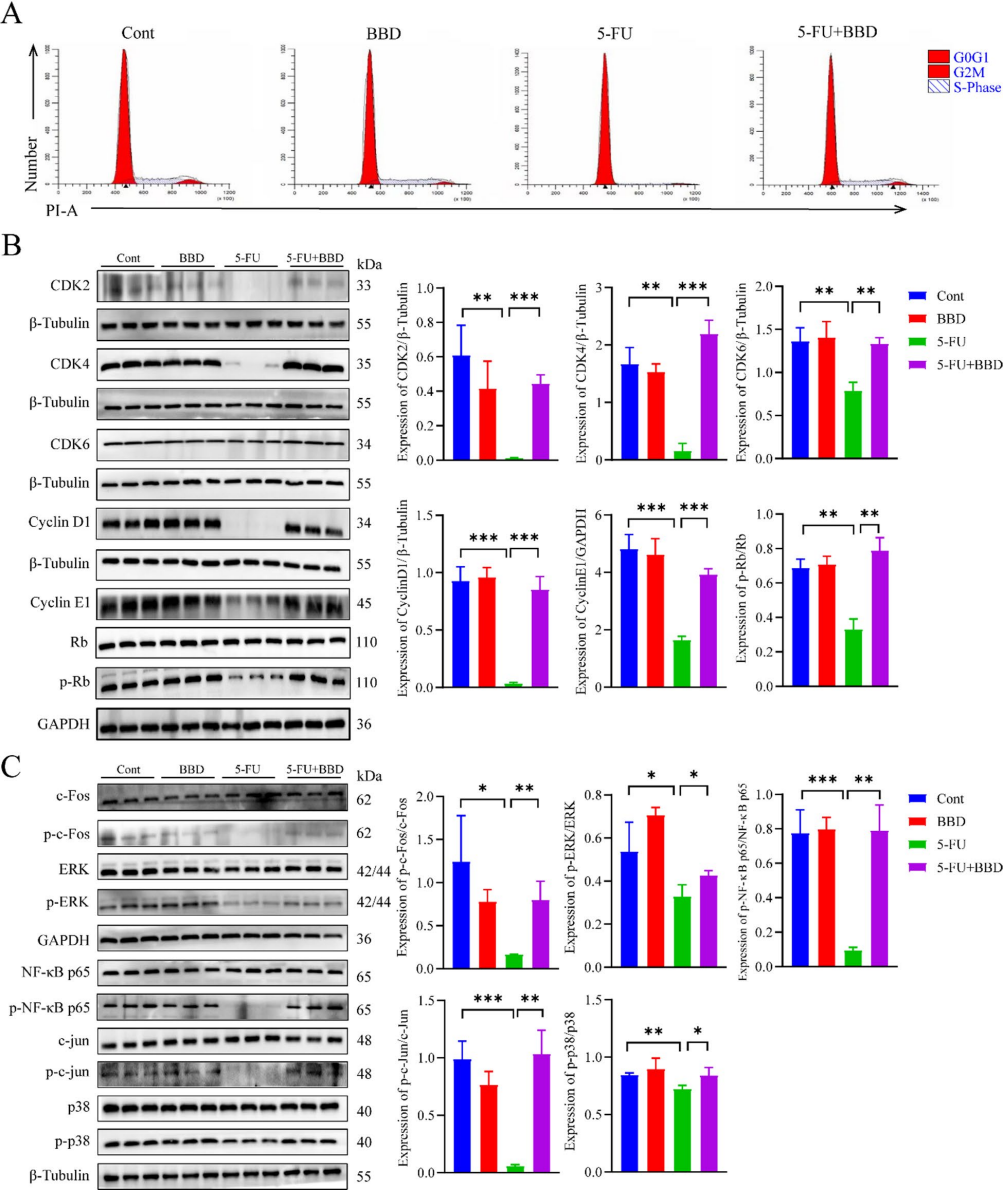
BBD attenuates 5-FU-induced cell cycle arrest by modulating the MAPK signaling pathway in myeloid cells. (**A**) Cell cycle of bone marrow cells. (**B**) The expression of cell cycle-related proteins. (**C**) The expression of MAPK signaling pathway-related proteins. Data were shown as mean ± S.D. *P < 0.05, **P < 0.01, and ***P < 0.001.

Given the critical role of the cell cycle in regulating cell proliferation and the MAPK signaling pathway’s influence on cell cycle progression, cell growth, survival, proliferation, and differentiation, we sought to elucidate whether the ameliorative effects of BBD on 5-FU-induced inhibition of cell activation and proliferation are mediated through the MAPK pathway. The subsequent experiments focused on the cell cycle of bone marrow cells and the expression of proteins associated with the cell cycle and the MAPK signaling pathway. Compared with the Cont group, mice in the 5-FU group exhibited a significant increase in the proportion of cells in the G0/G1 phase, along with a marked decrease in the proportions of cells in the G2/M and S phases. Conversely, compared with the 5-FU group, mice in the 5-FU + BBD group showed a significant reduction in the G0/G1 phase cell proportion and a notable increase in the proportions of cells in the G2/M and S phases (Fig. 10A; Table 9). These findings suggest that 5-FU induces cell cycle arrest in the G0/G1 phase, thereby inhibiting cell growth and proliferation. BBD, however, can mitigate the 5-FU-induced cell cycle arrest.

**Table 9:** Effects of BBD on the cell cycle of bone marrow cells from mice $(\overline {x} \pm s)$.

Group	G0/G1 (%)	G2/M (%)	S (%)
Cont	78.78 ± 1.52	3.680 ± 0.81	17.54 ± 0.72
BBD	76.17 ± 1.09	2.89 ± 0.54	20.94 ± 1.40*
5-FU	93.17 ± 0.47***	1.18 ± 0.32**	5.63 ± 0.47***
5-FU + BBD	78.15 ± 0.83^###^	3.26 ± 0.17^###^	18.59 ± 0.67^###^

**P<0.01 and ***P<0.001, compared with the Cont group; ^###^ *P*<0.001, compared with the 5-FU group.

CDK2, CDK4, CDK6, Cyclin D1, Cyclin E1, Rb, and p-Rb are key regulators of the cell cycle. Relative to the Cont group, the 5-FU group showed significantly decreased expression of CDK2, CDK4, CDK6, Cyclin D1, Cyclin E1, and p-Rb protein. In contrast, the 5-FU + BBD group exhibited increased expression of these proteins (Fig. 10B). These results indicate that 5-FU inhibits the expression of these cell cycle regulators, causing G1 phase arrest and suppressing cell proliferation. BBD, however, mitigates 5-FU-induced cell cycle arrest, restores normal cell cycle progression, and reinstates cell proliferation.

The proteins c-Fos, p-c-Fos, c-Jun, p-c-Jun, ERK, p-ERK, NF-κB p65, p-NF-κB p65, p38, and p-p38 play pivotal roles in modulating the MAPK signaling pathway. Compared with the Cont group, mice in the 5-FU group exhibited significantly reduced expression levels of p-c-Fos, p-c-Jun, p-ERK, p-NF-κB p65, and p-p38. Conversely, in the 5-FU + BBD group, the expression levels of these phosphorylated proteins were markedly increased (Fig. 10C). These findings suggest that 5-FU inhibits the phosphorylation of c-Fos, c-Jun, ERK, NF-κB p65, and p38, thereby suppressing the MAPK signaling pathway. In contrast, BBD upregulates the phosphorylation levels of these proteins inhibited by 5-FU, thereby activating the MAPK signaling pathway.

## Discussion

BBD, a state-protected TCM, demonstrates dual antitumor and chemoprotective properties, highlighting its potential for integrative cancer therapy^13,15,21^. HPLC-MS/MS analysis identified key bioactive constituents, including crotonoside, isofraxidin, diosgenin, and ginsenoside Rb1, which contribute to these dual effects. Specifically, isofraxidin inhibits colorectal cancer via Akt pathway blockade while preventing myelosuppression^22,23^. Diosgenin suppresses ovarian cancer and protects against cisplatin-induced toxicity through Nrf2/HO-1 modulation^24–26^. Ginsenoside Rb1 attenuates stress-induced colorectal cancer via glycolytic modulation, ameliorates myelosuppression, and protects against doxorubicin-induced cardiotoxicity by inhibiting autophagy/ferroptosis^27–29^. These findings position BBD’s components as a multifaceted therapeutic platform, offering both direct antitumor activity and comprehensive chemoprotection, warranting further investigation.

5-FU, a common chemotherapeutic agent, induces myelosuppression (leukopenia, bone marrow damage), gastrointestinal toxicity, and neurotoxicity. Severe myelosuppression often necessitates chemotherapy dose reduction or discontinuation, adversely affecting patient outcomes^30^. Previous studies have indicated that BBD can alleviate intestinal injury induced by 5-FU through activation of the Wnt/β-catenin signaling pathway^13,14^. In contrast, our study reveals that BBD can also mitigate 5-FU-induced myelosuppression and T-cell immune impairment by modulating the MAPK pathway. This multi-organ synergistic protective effect of BBD may originate from its characteristic “multi-component, multi-target” mode of action. For instance, some of its components may accumulate in the intestine, where they are more likely to act on targets regulating the Wnt/β-catenin pathway, while other components or their metabolites enter the systemic circulation and may preferentially regulate bone marrow hematopoiesis and immune function via the MAPK pathway. Such multi-pathway synergistic action enhances the body’s overall capacity to resist 5-FU toxicity. This represents a new dimension of BBD’s chemoprotective effects, shifting the focus from epithelial barrier repair to systemic hematopoietic and immune reconstitution.

In this study, BBD significantly improved mouse survival, attenuated body weight loss, and mitigated splenic atrophy, indicating its potential to preserve systemic and immune function post‑chemotherapy. Hematological analyses confirmed that 5‑FU preferentially suppressed the leukocyte lineage, particularly granulopoiesis—a pattern consistent with clinical 5‑FU‑induced myelosuppression. Importantly, BBD counteracted these deficits by promoting granulocyte and lymphocyte recovery, enhancing reticulocyte production, and restoring overall bone marrow proliferative activity. These myeloprotective effects may be attributed to active constituents of BBD, research has indicated that ginsenoside Rb1 significantly enhances hematopoietic function^31^. Furthermore, studies have corroborated the efficacy of diosgenin in ameliorating pancytopenia and symptoms of bone marrow failure, thereby augmenting the peripheral blood cell count in Aplastic Anemia (AA) mice and stimulating cell proliferation within the bone marrow cavity^32,33^.

To further evaluate the immunomodulatory effects of BBD, we analyzed leukocyte populations by flow cytometry. After undergoing 5-FU chemotherapy, a significant reduction in the proportions of CD45-positive cells and granulocytes was observed, indicating that 5-FU inhibits the relative abundance of leukocytes and granulocytes within the spleen. Conversely, BBD was found to upregulate the suppressed leukocyte proportion, which aligns with the findings from hematological examinations. Consequently, it can be inferred that BBD ameliorates the 5-FU-induced imbalance in leukocyte proportions. Previous studies have established that 5-FU elicits a decrease in leukocyte counts^34^, whereas diosgenin, a component of BBD, has been demonstrated to mitigate this abnormal leukocyte status^35^.

Following 5-FU chemotherapy, a decrease is observed in both the proportion of Th1 cells and the Th1/Th2 ratio, resulting in the manifestation of a Th1/Th2 immune drift phenomenon. This represents a common type of cellular immune dysregulation within the body, which can be corrected by BBD. Some experiments have shown that diosgenin may bolster the immune defense capabilities of the organism by upregulating the expression of Th1 cytokines^36,37^. Based on these findings, it is plausible that BBD, potentially via its constituent diosgenin, corrects the Th1/Th2 immune drift and ameliorates the immune suppression induced by 5-FU chemotherapy.

Our findings reveal a significant shift in T cell subset distribution following 5-FU chemotherapy, characterized by selective depletion of terminally differentiated effector memory T cells (both CD4^+^ and CD8^+^) and relative preservation of naïve T cells. This differential susceptibility likely stems from the heightened metabolic activity and proliferative potential of terminally differentiated effector cells, making them more vulnerable to 5-FU’s antimetabolite mechanism targeting rapidly dividing cells. Notably, BBD specifically restored the CD4^+^ terminally differentiated effector memory population while reducing naïve T cell proportions. In addition, 5-FU treatment significantly suppressed T cell activation, as evidenced by reduced expression of CD25 and CD69 in both CD4^+^ and CD8^+^ subsets. Importantly, our study further demonstrates that BBD counteracts 5-FU-mediated suppression of T cell activation.

Our study demonstrates that 5-FU significantly suppresses T-cell proliferation, as evidenced by reduced Ki67/PCNA expression in splenocytes and decreased cell division proportion in CFSE analysis. Importantly, BBD reversed these effects, restoring proliferation capacity. T-cell proliferation is intimately associated with the differentiation of various T-cell subsets. As 5-FU inhibits T-cell proliferation, it subsequently impedes T-cell differentiation, potentially leading to an imbalance in the proportions of T-cell subsets such as Th1, Treg, naive T cells, and terminally differentiated effector memory T cells. This disruption can hinder the ability of CD4^+^T cells and CD8^+^T cells to fulfill their roles in immune regulation and specific cytotoxicity, respectively. Our research has uncovered that BBD has the capacity to ameliorate the phenomenon of T-cell proliferation suppression induced by 5-FU. By effectively stimulating T-cell proliferation and differentiation, BBD rectifies the imbalanced state of T-cell subsets, thereby restoring the functionality of CD4^+^T cells and CD8^+^T cells. Compared to previous studies, our research uncovers a distinct role for BBD in the regulation of T‑cell immunity.

Cell cycle progression is essential for proliferation. Our study demonstrates that 5-FU induces G0/G1 phase arrest, inhibiting proliferation, while BBD counteracts this effect. Mechanistically, 5-FU downregulates CDK4/6, Cyclin D, CDK2, and Cyclin E, impairing complex formation and reducing p-Rb phosphorylation. This blocks the E2F pathway, stalling G1-to-S phase transition. BBD restores orderly cell cycle progression by mitigating these effects. Supporting evidence comes from studies showing diosgenin (a BBD component) promotes G0/G1-to-S phase transition in mouse testicular supporting cells^38^, aligning with our findings.

Cell cycle regulation involves multiple factors, including CDK4/6, which are important downstream targets of the MAPK signaling pathway. The cell cycle is regulated by the MAPK signaling pathway, which is closely related to T cell activation and influences T cell development, differentiation, proliferation, and function^39–42^. Studies have found that 5-FU exhibits varying degrees of inhibitory effects on the activation of ERK, JNK, and p38, thereby inhibiting the MAPK pathway and downregulating the expression of Cyclin D, Cyclin B, and CDK2. This leads to cell cycle arrest in the S phase, inhibiting cell proliferation and inducing cell apoptosis and senescence^43,44^. Our study reveals that 5-FU suppresses the MAPK pathway by reducing phosphorylation of c-Fos, c-Jun, ERK, NF-κB p65, and p38, whereas BBD restores their activation. Previous studies have shown that active ERK, a key MAPK signaling component, can phosphorylate transcription factors such as Elk‑1, thereby enhancing c‑Fos expression and AP‑1 activity, which directly upregulates Cyclin D1 transcription^45–48^. Additionally, ERK may promote Cyclin D1 expression through other effectors, such as SOX9, which binds to the Cyclin D1 promoter^49^. Based on these established transcriptional pathways, we propose that BBD‑activated MAPK signaling facilitates G1/S transition in cells. These pathways align with our observed upregulation of p‑ERK, p‑c‑Fos, and Cyclin D1 after BBD treatment. This suggests that BBD may modulate the MAPK signaling pathway to alleviate the suppression of T-cell proliferation and activation caused by 5-FU chemotherapy. Consistent with these findings, previous studies have demonstrated that ginsenoside Rb1 - a key component of BBD - enhances phosphorylation of p38 (MAPK)^50^. Additionally, it has been reported that ginsenoside Rb1 activates ERK1/2 through estrogen receptor stimulation^51^. Based on this evidence, it is hypothesized that ginsenoside Rb1 may contribute to BBD-mediated activation of the MAPK signaling pathway.

Despite the promising findings, this study has several limitations. First, the protective effects of BBD were observed in an acute 5-FU model, which may not fully reflect the chronic bone marrow injury in clinical settings. Second, while we identified the involvement of the MAPK pathway, the multi-component nature of BBD suggests that other signaling pathways likely contribute to its holistic effects, warranting further multi-omics exploration. Finally, while our evaluation of immune status is extensive in profiling T cell subsets, activation markers, and proliferative capacity, it still lacks a comprehensive validation of “overall immune homeostasis.” Addressing these limitations in future research would facilitate the translation of BBD into a standardized, evidence-supported therapy for tumor chemotherapy.

## Conclusion

**Fig. 11:**
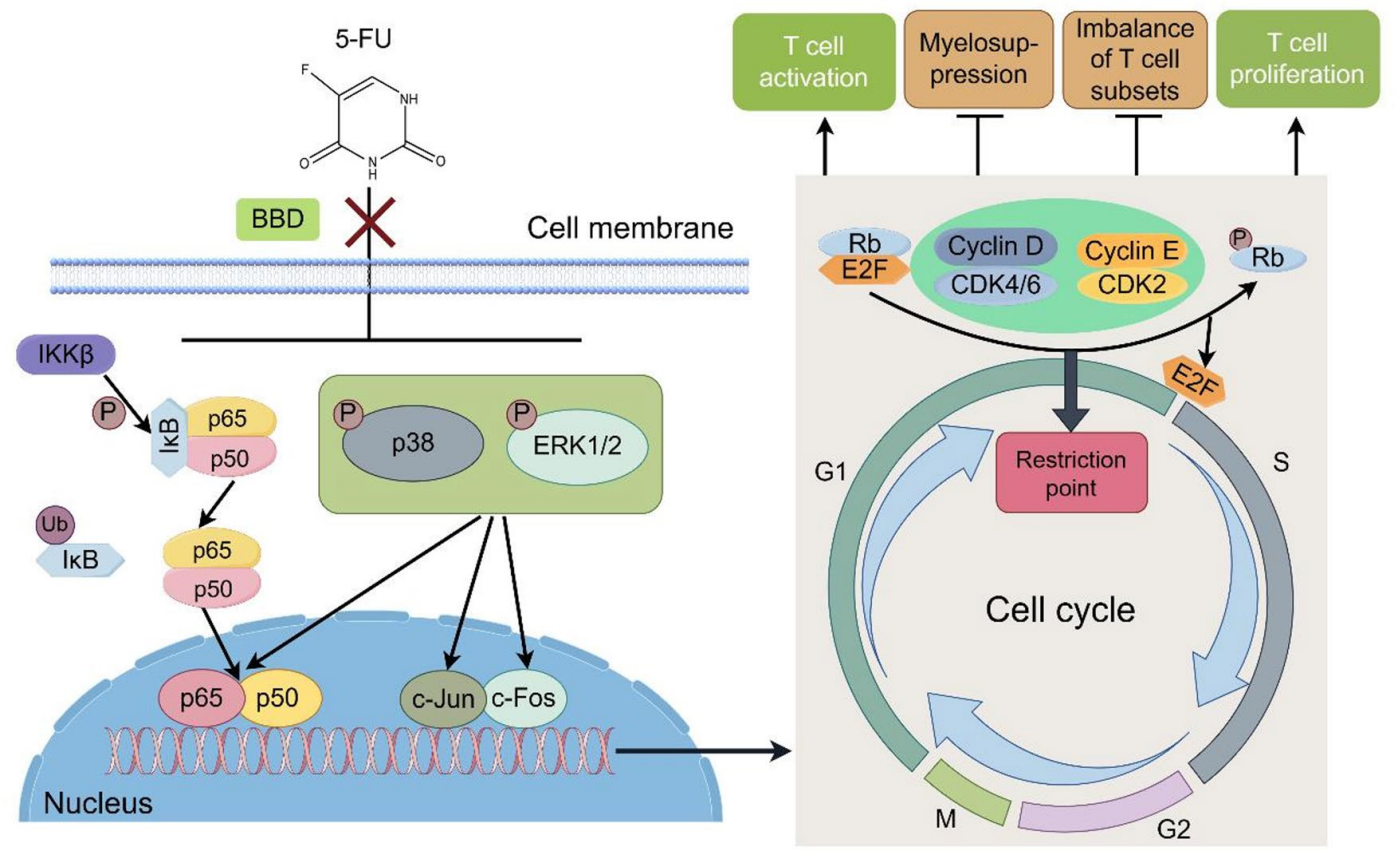
The mechanism by which Babao Dan attenuates fluorouracil-induced bone marrow suppression and T cell homeostasis disruption.

In conclusion, BBD has been observed to enhance the survival rate of mice undergoing 5-FU chemotherapy, alleviating symptoms such as weight loss, spleen atrophy, leukopenia, decreased bone marrow hematopoiesis, inhibited granulocyte differentiation, and imbalances in T cell subpopulations. Further investigation suggests that BBD promotes bone marrow proliferation and ameliorates T cell immune dysregulation, potentially by regulating the MAPK signaling pathway. This regulation enhances the expression of G1/S checkpoint-related proteins in bone marrow cells, upregulates the phosphorylation level of Rb, facilitates the smooth transition of cells from the G1 phase to the S phase, promotes cell proliferation, and restores the hematopoietic function of bone marrow cells after 5-FU chemotherapy. Consequently, this improves the ratio of peripheral T cell subpopulations, corrects the imbalance in T cell subpopulations, restores the activation and proliferation functions of CD4^+^T cells and CD8^+^T cells, and ameliorates the state of immune dysregulation in the body (Fig. 11).

## Data Availability

The datasets used and/or analysed during the current study available from the corresponding author on reasonable request.

## Abbreviations

5-FU: 5-Fluorouracil
BBD: Babao Dan
CDK2: Cyclin-dependent kinases 2
CDK4: Cyclin-dependent kinases 4
CDK6: Cyclin-dependent kinases 6
CDKI: Cyclin-dependent kinase inhibitor
c-Fos: Cellular FBJ osteosarcoma oncogene protein
CFSE: Carboxyfluorescein Succinimidyl Ester
c-Jun: Cellular Jun proto-oncogene protein
ERK: Extracellular regulated protein kinases
HCT: Hematocrit
HFR: Percentage of high fluorescence intensity reticulocytes
HGB: Hemoglobin
HPCs: Hematopoietic progenitor cells
HSCs: Hematopoietic stem cells
IFN-γ: Interferon-γ
IHC: Immunohistochemistry
IL-2: Interleukin-2
IL-7: Interleukin-7
IL-12: Interleukin-12
IL-21: Interleukin-21
IRF: Percentage of immature reticulocytes
Ki67: Kiel 67 protein
LC-MS/MS: Liquid Chromatography-Tandem Mass Spectrometry
LFR: Percentage of low fluorescence intensity reticulocytes
LYM: The percent of lymphocyte
MAPK: Mitogen-activated protein kinase
MFR: Percentage of medium fluorescence intensity reticulocytes
MONO: The percent of monocyte
MPV: Mean platelet volume
NEUT: Absolute neutrophil count
NF-κB: Nuclear factor kappa-B
PCNA: Proliferating Cell Nuclear Antigen
PCT: Plateletcrit
PDW: Platelet distribution width
PLT: Platelet count
Rb: Retinoblastoma protein
RBC: Red blood cell count
RDW: Red cell volume distribution width
RET: Reticulocyte
Th: Helper T
TNF-α: Tumor necrosis factor-α
Treg: Regulatory T cells
WBC: White blood cell count

## References

[1] Han, B. et al. Cancer incidence and mortality in China, 2022. *J. Natl. Cancer Cent.* **4** (1), 47–53 (2024). 39036382 10.1016/j.jncc.2024.01.006 PMC11256708

[2] Barreto, J. N., McCullough, K. B., Ice, L. L. & Smith, J. A. Antineoplastic agents and the associated myelosuppressive effects:a review. *J. Pharm. Pract.* **27** (5), 440–446 (2014). 25147158 10.1177/0897190014546108

[3] Hoekman, K. et al. Effects of Recombinant human granulocyte-macrophage colony-stimulating factor on myelosuppression induced by multiple cycles of high-dose chemotherapy in patients with advanced breast cancer. *J. Natl. Cancer Inst.* **83** (21), 1546–1553 (1991). 1960751 10.1093/jnci/83.21.1546

[4] Molyneux, G. et al. Haemotoxicity of busulphan, doxorubicin, cisplatin and cyclophosphamide in the female BALB/c mouse using a brief regimen of drug administration. *Cell. Biol. Toxicol.* **27** (1), 13–40 (2011). 20589437 10.1007/s10565-010-9167-1

[5] Le Rhun, E. et al. Prognostic significance of therapy-induced myelosuppression in newly diagnosed glioblastoma. *Neuro Oncol.* **24** (9), 1533–1545 (2022). 35312789 10.1093/neuonc/noac070 PMC9435483

[6] Li, Y., Wu, Z., Ni, C., Li, Y. & Wang, P. Evaluation of the clinical significance of lymphocyte subsets and myeloid suppressor cells in patients with renal carcinoma. *Discov Oncol.* **15** (1), 512 (2024). 39347882 10.1007/s12672-024-01405-2 PMC11442913

[7] Dixon-Douglas, J. et al. Sustained lymphocyte decreases after treatment for early breast cancer. *NPJ Breast Cancer*. **10** (1), 94 (2024). 39433772 10.1038/s41523-024-00698-4 PMC11493948

[8] Chapman, N. M., Boothby, M. R. & Chi, H. Metabolic coordination of T cell quiescence and activation. *Nat. Rev. Immunol.* **20** (1), 55–70 (2020). 31406325 10.1038/s41577-019-0203-y

[9] Tu, H. F. et al. FLT3L-induced virtual memory CD8 T cells engage the immune system against tumors. *J. Biomed. Sci.* **31** (1), 19 (2024). 38287325 10.1186/s12929-024-01006-9 PMC10826030

[10] Reina-Campos, M., Scharping, N. E. & Goldrath, A. W. CD8(+) T cell metabolism in infection and cancer. *Nat. Rev. Immunol.* **21** (11), 718–738 (2021). 33981085 10.1038/s41577-021-00537-8 PMC8806153

[11] Sharabi, A. & Tsokos, G. C. T cell metabolism: new insights in systemic lupus erythematosus pathogenesis and therapy. *Nat. Rev. Rheumatol.* **16** (2), 100–112 (2020). 31949287 10.1038/s41584-019-0356-x

[12] Song, L. B., Gao, S., Zhang, A. Q., Qian, X. & Liu, L. M. Babaodan capsule () combined with Qingyi Huaji formula () in advanced pancreatic cancer-a feasibility study. *Chin. J. Integr. Med.* **23** (12), 937–942 (2017). 28664246 10.1007/s11655-017-2279-1

[13] Gui, M. X. et al. Babao Dan alleviates 5-Fluorouracil-Induced intestinal damage via Wnt/β-Catenin pathway. *Chin. J. Integr. Med.* **28** (11), 1000–1006 (2022). 33420580 10.1007/s11655-021-3282-0

[14] Huang, B. et al. Babao Dan alleviates gut immune and microbiota disorders while impacting the TLR4/MyD88/NF-кB pathway to attenuate 5-Fluorouracil-induced intestinal injury. *Biomed. Pharmacother*. **166**, 115387 (2023). 10.1016/j.biopha.2023.115387 37643486

[15] Qiu, Z. et al. Babao Dan alleviates cancer cachexia in mice via inhibiting IL-6/STAT3 signaling pathway. *Integr. Cancer Ther.* **22**, 15347354231168369 (2023). 37077153 10.1177/15347354231168369 PMC10126781

[16] Xie, X. et al. Babao Dan is a robust anti-tumor agent via inhibiting wnt/β-catenin activation and cancer cell stemness. *J. Ethnopharmacol.* **280**, 114449 (2021). 34332067 10.1016/j.jep.2021.114449

[17] Guan, J. et al. Babao Dan inhibits lymphangiogenesis of gastric cancer in vitro and in vivo via lncRNA-ANRIL/VEGF-C/VEGFR-3 signaling axis. *Biomed. Pharmacother*. **154**, 113630 (2022). 36058147 10.1016/j.biopha.2022.113630

[18] Liu, J. et al. Babao Dan inhibits the migration and invasion of gastric cancer cells by suppressing epithelial-mesenchymal transition through the TGF-β/Smad pathway. *J. Int. Med. Res.* **48** (6), 300060520925598 (2020). 32529872 10.1177/0300060520925598 PMC7294507

[19] Cibrian, D. & Sanchez-Madrid, F. CD69: from activation marker to metabolic gatekeeper. *Eur. J. Immunol.* **47** (6), 946–953 (2017). 28475283 10.1002/eji.201646837 PMC6485631

[20] Shipkova, M. & Wieland, E. Surface markers of lymphocyte activation and markers of cell proliferation. *Clin. Chim. Acta*. **413** (17–18), 1338–1349 (2012). 22120733 10.1016/j.cca.2011.11.006

[21] Zhao, J. et al. Babao Dan reverses Multiple-Drug resistance in gastric cancer cells via triggering apoptosis and autophagy and inhibiting PI3K/AKT/mTOR signaling. *Evid. Based Complement. Alternat Med.* **2021**, 5631942 (2021). 34306145 10.1155/2021/5631942 PMC8285167

[22] Shen, P. et al. Isofraxidin inhibited proliferation and induced apoptosis via blockage of Akt pathway in human colorectal cancer cells. *Biomed. Pharmacother*. **92**, 78–85 (2017). 28531803 10.1016/j.biopha.2017.05.065

[23] Wang, C. et al. Protective effects of acanthopanax senticosus - Ligustrum lucidum combination on bone marrow suppression induced by chemotherapy in mice. *Biomed. Pharmacother*. **109**, 2062–2069 (2019). 30551462 10.1016/j.biopha.2018.11.071

[24] Wang, S. et al. Dioscin exerts nephroprotective effects by attenuating oxidative stress and necroptosis-induced inflammation. *Int. Immunopharmacol.* **140**, 112885 (2024). 39116496 10.1016/j.intimp.2024.112885

[25] Jin, S. et al. Dioscin ameliorates cisplatin-induced intestinal toxicity by mitigating oxidative stress and inflammation. *Int. Immunopharmacol.* **111**, 109111 (2022). 35933746 10.1016/j.intimp.2022.109111

[26] Fang, F., Zhang, X. & Fang, Y. Diosgenin inhibits proliferation and migration of ovarian cancer cells and induce apoptosis via upregulation of PTEN. *Chem. Biol. Drug Des.* **103** (3), e14459 (2024). 38538058 10.1111/cbdd.14459

[27] Han, J. et al. Compatibility effects of ginseng and ligustrum lucidum Ait herb pair on hematopoietic recovery in mice with cyclophosphamide-induced myelosuppression and its material basis. *J. Ginseng Res.* **44** (2), 291–299 (2020). 32148411 10.1016/j.jgr.2019.01.001 PMC7031745

[28] Zhai, Y. et al. Ginsenoside Rb1 attenuates doxorubicin induced cardiotoxicity by suppressing autophagy and ferroptosis. *Biochem. Biophys. Res. Commun.* **710**, 149910 (2024). 38593619 10.1016/j.bbrc.2024.149910

[29] Yao, W. et al. Ginsenoside Rb1 inhibits chronic stress-induced colorectal cancer via regulating Glycolysis and β2-AR/CREB1 signaling pathway. *J. Pharm. Pharmacol.* (2025). 10.1093/jpp/rgaf031 40498670

[30] Santos, C., Morgan, B. W. & Geller, R. J. The successful treatment of 5-fluorouracil (5-FU) overdose in a patient with malignancy and HIV/AIDS with uridine triacetate. *Am. J. Emerg. Med.* **35** (5), 802.e7–802.e8 (2017). 27884585 10.1016/j.ajem.2016.11.038

[31] Zhang, L. et al. Ginseng saponin Rb1 enhances hematopoietic function and dendritic cells differentiation. *Acta Biochim. Biophys. Sin (Shanghai)*. **49** (8), 746–749 (2017). 28655146 10.1093/abbs/gmx062

[32] Fan, L. et al. Dioscin alleviates aplastic anemia through regulatory T cells promotion. *Hematology* **29** (1), 2326389 (2024). 38466633 10.1080/16078454.2024.2326389

[33] Zhang, L. et al. Dioscin regulating bone marrow apoptosis in aplastic anemia. *Drug Des. Devel Ther.* **16**, 3041–3053 (2022). 36105320 10.2147/DDDT.S370506 PMC9467696

[34] Kobuchi, S. et al. Semi-physiological pharmacokinetic-pharmacodynamic modeling and simulation of 5-fluorouracil for the whole time course of alterations in leukocyte, neutrophil and lymphocyte counts in rats. *Xenobiotica* **44** (9), 804–818 (2014). 24650147 10.3109/00498254.2014.900588

[35] Semwal, P. et al. Diosgenin: an updated Pharmacological review and therapeutic perspectives. *Oxid. Med. Cell. Longev.* **2022**, 1035441 (2022). 35677108 10.1155/2022/1035441 PMC9168095

[36] Jan, T. R., Wey, S. P., Kuan, C. C., Liao, M. H. & Wu, H. Y. Diosgenin, a steroidal sapogenin, enhances antigen-specific IgG2a and interferon-gamma expression in ovalbumin-sensitized BALB/c mice. *Planta Med.* **73** (5), 421–426 (2007). 17566144 10.1055/s-2007-967169

[37] Huang, C. H., Wang, C. C., Lin, Y. C., Hori, M. & Jan, T. R. Oral administration with Diosgenin enhances the induction of intestinal T helper 1-like regulatory T cells in a murine model of food allergy. *Int. Immunopharmacol.* **42**, 59–66 (2017). 27886644 10.1016/j.intimp.2016.11.021

[38] Wu, L. et al. Diosgenin stimulates rat TM4 cell proliferation through activating plasma membrane translocation and transcriptional activity of Estrogen receptors. *Biol. Reprod.* **92** (1), 24 (2015). 25429088 10.1095/biolreprod.114.124206

[39] Dennison, L., Mohan, A. A. & Yarchoan, M. Tumor and systemic Immunomodulatory effects of MEK Inhibition. *Curr. Oncol. Rep.* **23** (2), 23 (2021). 33547983 10.1007/s11912-020-01008-4 PMC8028056

[40] Smith, T. et al. Peripheral deletion of CD8 T cells requires p38 MAPK in Cross-Presenting dendritic cells. *J. Immunol.* **199** (8), 2713–2720 (2017). 28864471 10.4049/jimmunol.1700427 PMC5679299

[41] Wei, X. et al. The evolutionarily conserved MAPK/Erk signaling promotes ancestral T-cell immunity in fish via c-Myc-mediated Glycolysis. *J. Biol. Chem.* **295** (10), 3000–3016 (2020). 31996375 10.1074/jbc.RA119.012231 PMC7062195

[42] Tong, C. et al. Forsythiaside a plays an anti-inflammatory role in LPS-induced mastitis in a mouse model by modulating the MAPK and NF-κB signaling pathways. *Res. Vet. Sci.* **136**, 390–395 (2021). 33799169 10.1016/j.rvsc.2021.03.020

[43] Zhao, J. et al. 5-fluorouracil suppresses stem cell-like properties by inhibiting p38 in pancreatic cancer cell line PANC-1. *Folia Histochem. Cytobiol*. **60** (1), 55–65 (2022). 35103981 10.5603/FHC.a2022.0004

[44] Li, H. et al. 5-Fluorouracil enhances the chemosensitivity of gastric cancer to TRAIL via Inhibition of the MAPK pathway. *Biochem. Biophys. Res. Commun.* **540**, 108–115 (2021). 33476960 10.1016/j.bbrc.2021.01.006

[45] Chambard, J. C., Lefloch, R., Pouysségur, J. & Lenormand, P. ERK implication in cell cycle regulation. *Biochim. Biophys. Acta*. **1773** (8), 1299–1310 (2007). 17188374 10.1016/j.bbamcr.2006.11.010

[46] Gao, F. et al. Xanthohumol targets the ERK1/2–Fra1 signaling axis to reduce Cyclin D1 expression and inhibit non–small cell lung cancer. *Oncol. Rep.* **44** (4), 1365–1374 (2020). 32945473 10.3892/or.2020.7697 PMC7448415

[47] Dumesic, P. A., Scholl, F. A., Barragan, D. I. & Khavari, P. A. Erk1/2 MAP kinases are required for epidermal G2/M progression. *J. Cell Biol.* **185** (3), 409–422 (2009). 19414607 10.1083/jcb.200804038 PMC2700391

[48] Sen, A. et al. Paxillin mediates extranuclear and intranuclear signaling in prostate cancer proliferation. *J. Clin. Investig.* **122** (7), 2469–2481 (2012). 22684108 10.1172/JCI62044 PMC3386821

[49] Xie, M. et al. Sublytic C5b-9 induces glomerular mesangial cell proliferation via ERK1/2-dependent SOX9 phosphorylation and acetylation by enhancing Cyclin D1 in rat Thy-1 nephritis. *Exp. Mol. Med.* **53** (4), 572–590 (2021). 33811247 10.1038/s12276-021-00589-9 PMC8102557

[50] Xin, C. et al. Ginsenoside Rb1 increases macrophage phagocytosis through p38 mitogen-activated protein kinase/Akt pathway. *J. Ginseng Res.* **43** (3), 394–401 (2019). 31308811 10.1016/j.jgr.2018.05.003 PMC6606816

[51] Hashimoto, R., Yu, J., Koizumi, H., Ouchi, Y. & Okabe, T. Ginsenoside Rb1 prevents MPP(+)-Induced apoptosis in PC12 cells by stimulating Estrogen receptors with consequent activation of ERK1/2, Akt and Inhibition of SAPK/JNK, p38 MAPK. *Evid. Based Complement. Alternat Med.* **2012**, 693717 (2012). 23024694 10.1155/2012/693717 PMC3457685

